# Supplementary material for: The Asgard archaeal ESCRT-III system forms helical filaments and remodels eukaryotic-like membranes
Source: EMBO J. 2025 Jan 3;44(3):665–81. doi: 10.1038/s44318-024-00346-4 (PMC11791191; doi:10.1038/s44318-024-00346-4)
Supplement: Supplementary file 1 — Appendix [file 44318_2024_346_MOESM1_ESM.pdf]

Appendix

The Asgard archaeal ESCRT-III system forms helical filaments and remodels eukaryotic-like membranes

Nataly Melnikov[1,2], Benedikt Junglas[3$], Gal Halbi[4$], Dikla Nachmias[1,2], Erez Zerbib[1,2], Noam Gueta[1,2], Alexander Upcher[5], Ran Zalk[5], Carsten Sachse[3,6*], Ann Bernheim-Groswasser[4,5], Natalie Elia[1,2*]

Table of contents

Appendix Figure S1 3
Appendix Figure S2 4
Appendix Figure S3 6
Appendix Figure S4 8
Appendix Figure S5 10
Appendix Figure S6 12
Appendix Figure S7 14
Appendix Figure S8 16
References 18

Appendix Figure S1

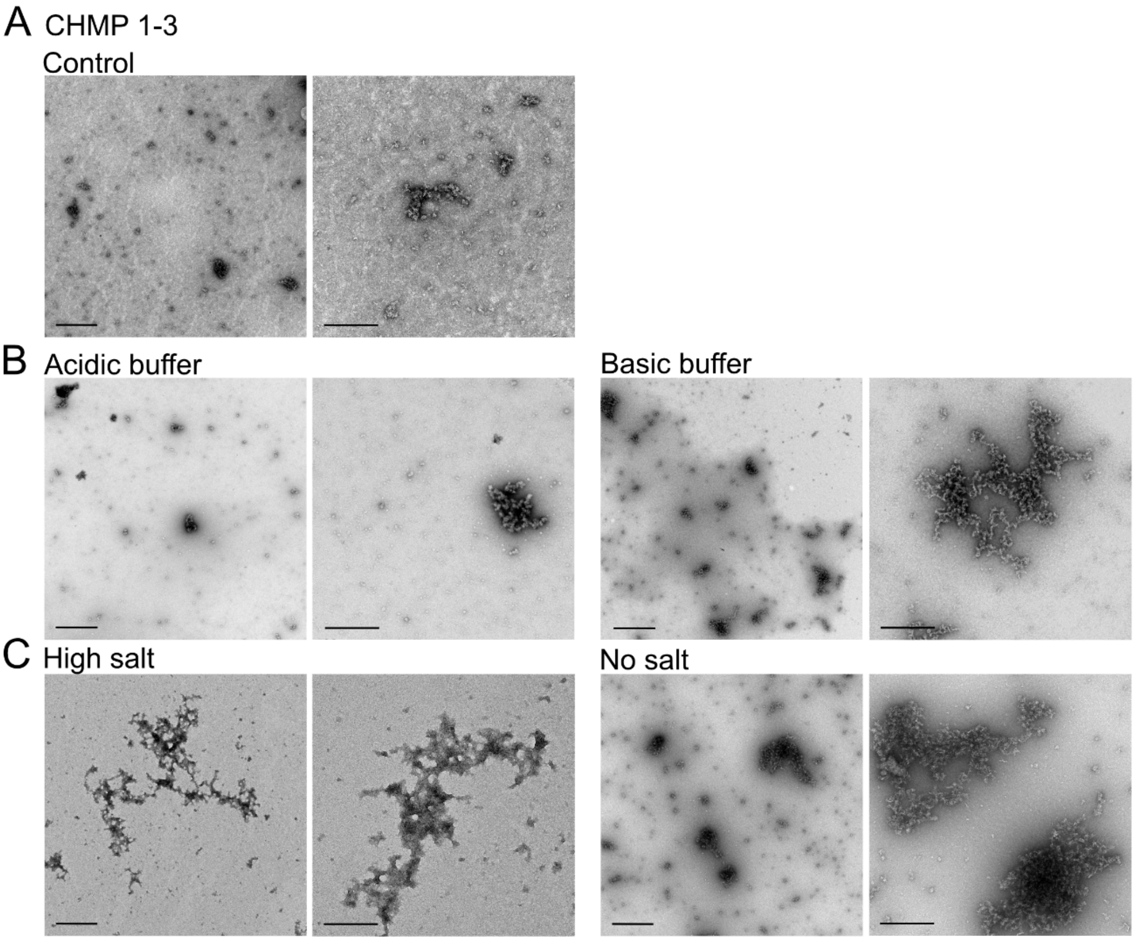

Appendix Figure S1: Loki CHMP1-3 do not self-assemble into high-ordered structures. (**A-C**) representative negative stain TEM images of full-length Loki CHMP1-3 that was subjected to polymerization reaction at different buffers. (**A**) buffer used for CHMP4-7 self-assembly: 25 mM Tris pH=7.6, 50 mM KCl). (**B**) Using acidic (50 mM Sodium Acetate pH=5, 50 mM KCl) or basic (25 mM Tris pH=8.8 50 mM KCl buffers). (**C**) at different ionic strengths (high salt: 25 mM Tris pH=7.6, 200 mM KCl; No salt: 25 mM Tris pH=7.6. A final concentration of 10.88 μM CHMP1-3 was used for all buffer conditions. No filaments were observed under any of the examined conditions. Data in all panels was reproduced in at least two independent experiments.

Appendix Figure S2

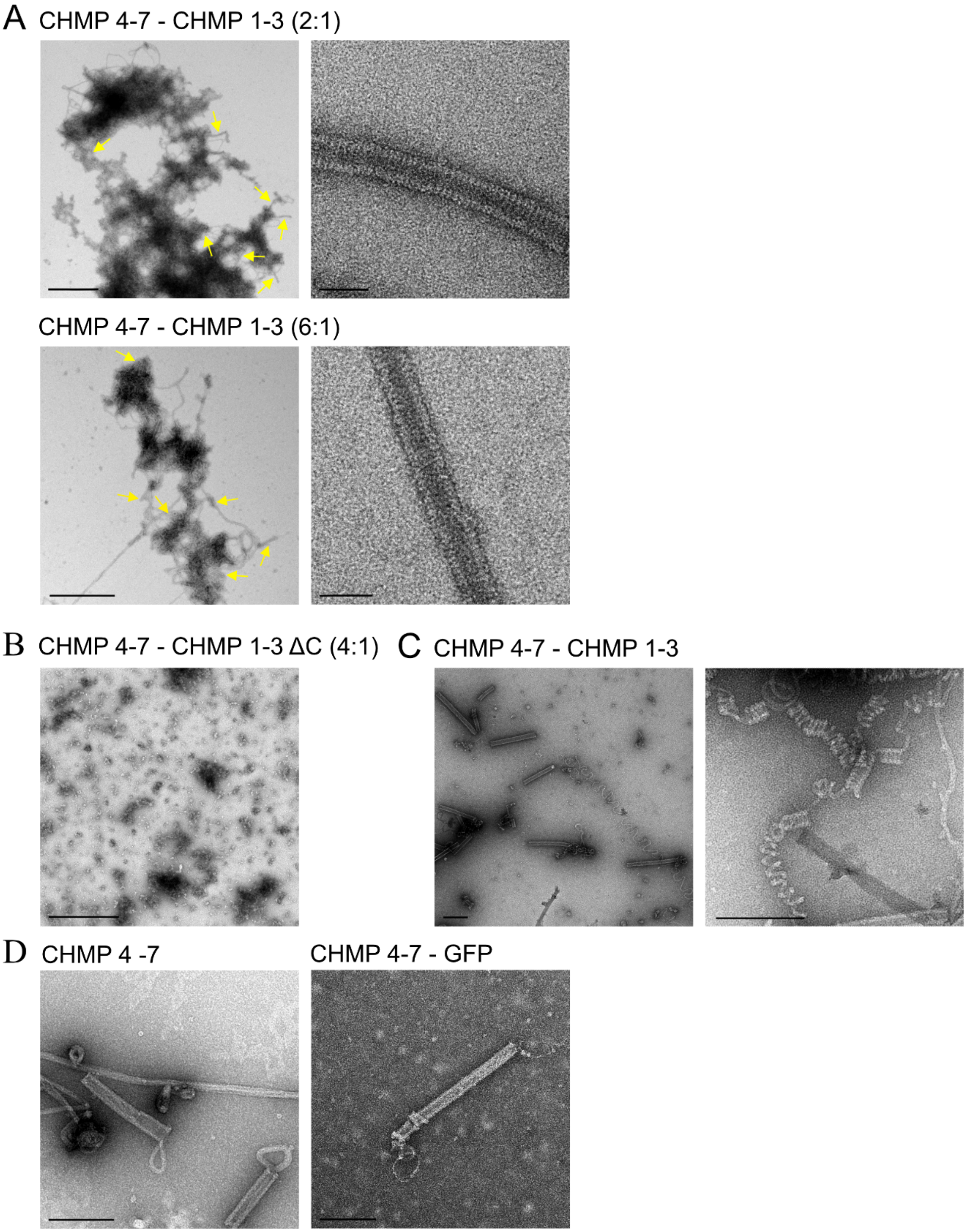

Appendix Figure S2: Characterization of Loki ESCRT-III filaments. A. Representative negative stain TEM images of Loki ESCRT-III filaments assembled using CHMP4-7 and CHMP1-3 at molar ratios 2:1 (upper panel) and 6:1 (bottom panel). Zoomed-out (left, Scale = 1 μm); zoomed-in (right, Scale = 50 nm). Yellow arrows indicate helical tubes. Corresponds to measurements in Fig. 2B. **B.** Negative stain TEM images obtained using CHMP4-7 and CHMP1-3 C' deletion mutant (AAs 1-163) at molar ratios 4:1. No filaments were observed under these conditions. Scale = 1 μm. **C.** Negative stain TEM images of loose Loki Loki ESCRT-III filaments assembled in the presence of CHMP4-7 – CHMP1-3 (2:1 molar ratio, ssDNA) resembling a telephone cord, supporting that the ESCRT-III tube is composed of a packed helical filament. Scale = 200 nm **D.** Representative negative stain TEM images of filaments assembled in the presence of Loki CHMP 4-7 at a total concentration that equals the concentration of both CHMP4-7 - CHMP1-3 at 2:1 ratio (left, final concentration of 16 μM) and in the presence of both CHMP4-7 and purified recombinant GFP (right panel), reaching the same final concentrations (CHMP 4-7 10.67 μM, GFP 5.4 μM). Scale = 200 nm. No effect on filament formation was identified under these assembly conditions. Data in all panels was reproduced in at least two independent experiments.

Appendix Figure S3

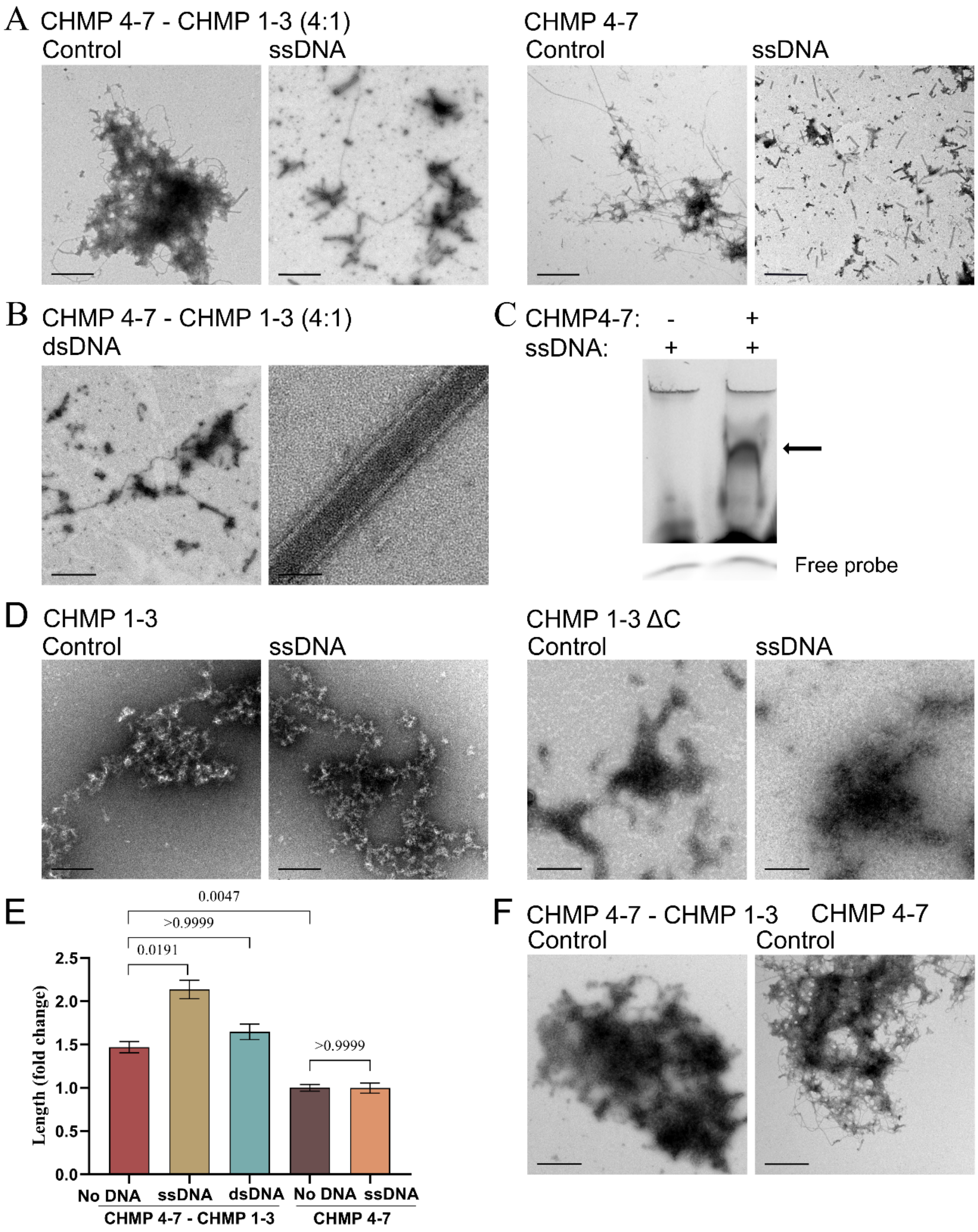

Appendix Figure S3: Helical tube formation by Loki ESCRT-III in the presence of nucleic acids. A. Raw data of the representative zoomed-out negative stain TEM images presented in Fig. 3A. Scale = 1 µm. **B.** CHMP4-7 and CHMP1-3 (4:1 molar ratio) were subjected to polymerization reaction (see methods) in the presence of 40 bases dsDNA, and documented using negative-stain TEM. Scale zoomed out, 1 µm; zoomed in, 50 nm. **C.** Electrophoretic mobility shift assay (EMSA) performed in the presence of Cy5-5' end labeled ssDNA probe (2.5 µM, 40 bases) and the purified Loki CHMP4-7 protein - 5 µl (see methods (Nachmias *et al*, 2022). Arrows in upper panel indicate complex formation. Lower panel, free DNA probe as seen by shorter exposure time.

D. Representative zoomed-out negative stain TEM images of assembly reactions using Loki CHMP 1–3 (full-length or C' deletion mutant) with or without ssDNA (40 bases probe) showing that CHMP 1-3 does not polymerize under any of those conditions. Scale = 200 nm. **E.** Length measurements of helical tubes formed under the indicated conditions (corresponds to Fig. 3). Measurements obtained from negative stain TEM images of samples produced in at least two independent experiments for each condition. Averaged lengths (nm): CHMP4-7/CHMP1-3 534.8 ±118.8, n=25; CHMP4-7/CHMP1-3 ssDNA 777.5 ±210.6, n=30; CHMP4-7/CHMP1-3 dsDNA 599±218.2, n=45; CHMP4-7 364±67.8, n=23; CHMP4-7 ssDNA 363 ± 96.1, n = 21. Statistics was performed using one-way analysis of variance (ANOVA) (see methods). **F.** Raw data of the representative zoomed-out negative stain TEM images presented in Fig. 3E.

Appendix Figure S4

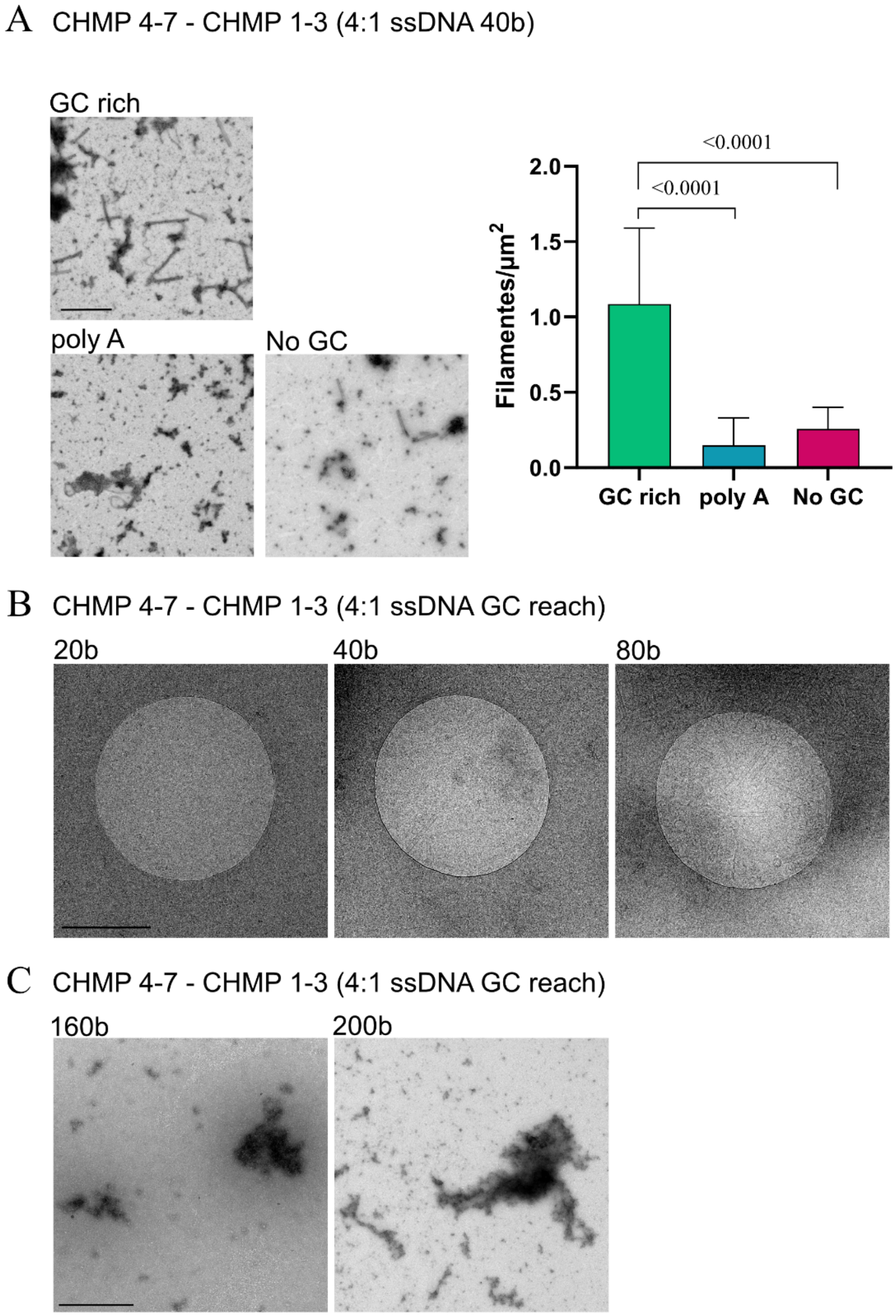

Appendix Figure S4: Characterization of oligonucleotides compositions that induces Loki ESCRT-III polymerization

A. Representative zoomed-out negative stain TEM images obtained for Loki CHMP 4-7 – CHMP 1-3 (4:1) protein samples that were assembled in the presence of 40 bases long ssDNAs comprising different sequences (10 μM): GC rich – the typical ssDNA probe used in this study (see methods section); No GC – AATAAATTATTTAAATAAATATAAATTAAATAAATTATAA; Poly A – a 40 bases long sequence comprised of only the nucleotide A. Quantification of Helical tubes density at various conditions is shown on the right. Measurements were performed on an area > 600 μm^2 for each condition. Oligonucleotides without GC do not induce polymerization. Statistics was performed using one-way analysis of variance (ANOVA) (see methods).Scale = 1 μm. **B.** Representative zoomed-out Cryo EM images obtained for Loki CHMP 4-7 – CHMP 1-3 (4:1) protein samples that were assembled in the presence of ssDNA probes of different lengths, as indicated. ssDNA 20b (GACCCGTTTAGAGGCCCCAA); ssDNA 40b (as in method section); ssDNA 80b (GTCTGGTGCCACGCGGTAGTGGTGGTATCGAAGGTAGGCAGGAGAATCTGTACT TTCAGGGCGCTAGCCATATGTCATCG). Note that filaments could be readily observed in the presence of 40 and 80 bases long ssDNA, but not in the presence of a 20-nucleotide probe. Scale = 600 nm. **C.** Representative zoomed-out negative stain TEM images obtained for Loki CHMP 4-7 – CHMP 1-3 (4:1) protein samples that were assembled in the presence of 160, 200 bases long ssDNAs comprising 4-5 repetitions of the typical 40 bases long used in this study (see methods section). No filaments were observed under these conditions. Scale = 1 μm.

Appendix Figure S5

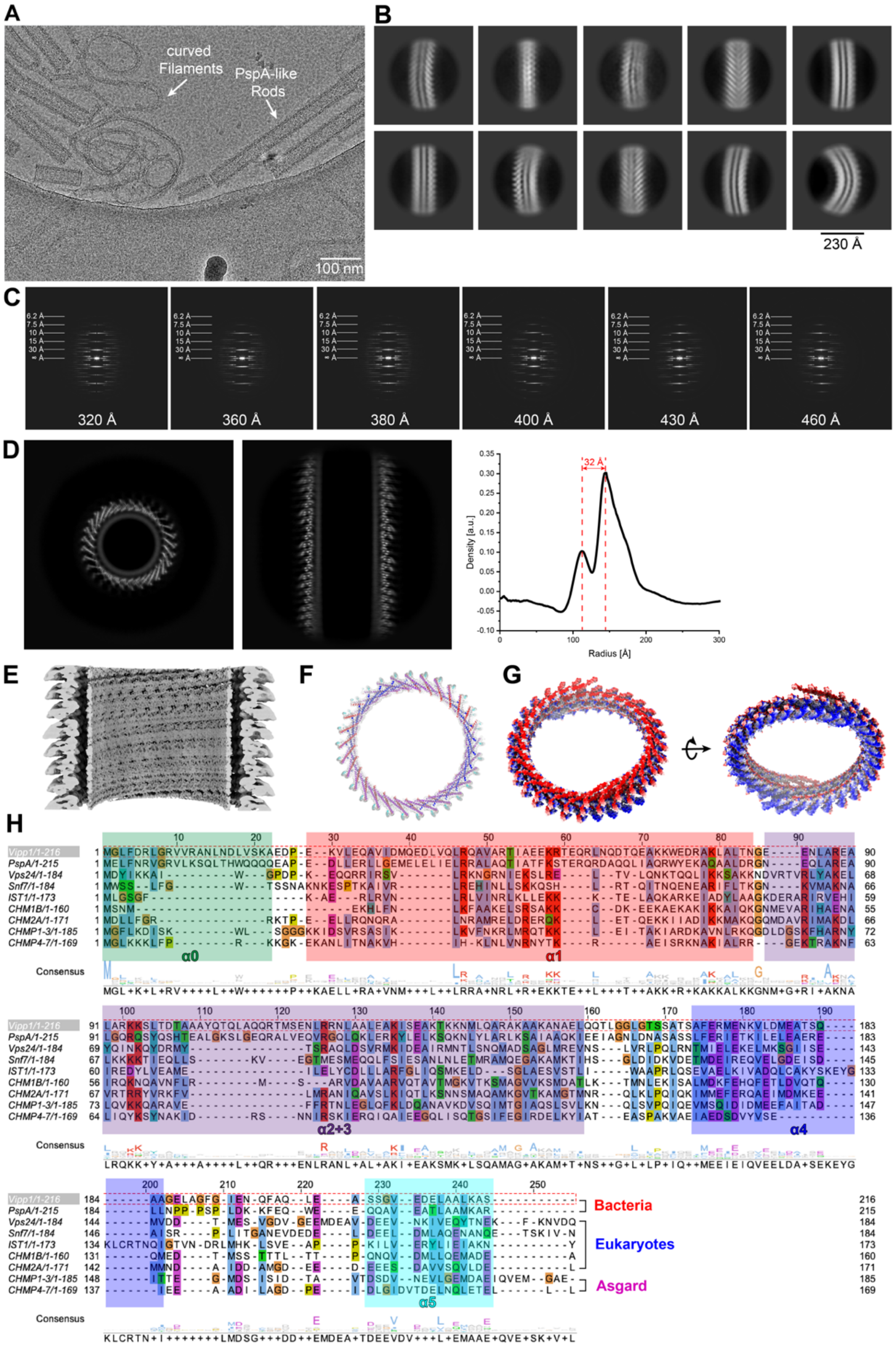

Appendix Figure S5: Curved Filaments and ESCRT-III comparison.

A. Cryo-EM micrograph of CHMP 4-7/CHMP 1-3 (4:1 molar ratio, assembled in the presence of ssDNA) showing curved filaments and PspA-like rods. **B.** 2D class averages of curved filaments**. C.** Power spectra of 2D class averages with diameters of 320, 340, 360, 380, 400 and 420 Å, respectively. As the layer line heights and overall pattern are very similar, the assemblies vary only in the additional units per turn. **D.** z and xy slice (averaged over 10 px) of the Loki CHMP4-7 rods showing tube-like fuzzy density in the lumen of the rods. **D.** Top view of the density map and the fitted polymer model. **E.** Electrostatic surface of helical assembly reveals a negatively and a positively charged end indicating a distinct polarity of the rod. **F.** Sequence alignment of ESCRT-III proteins with known structures (truncated to α5) in comparison with Loki CHMP 1-3 and 4-7. The alignment is superimposed with the consensus secondary structures derived from the deposited models. Residues with similarity >30% are colored according to their properties (ClustalX color scheme). Sequences were aligned using the T-Coffee webserver and visualized with JalView (Di Tommaso *et al*, 2011; Waterhouse *et al*, 2009).

Appendix Figure S6

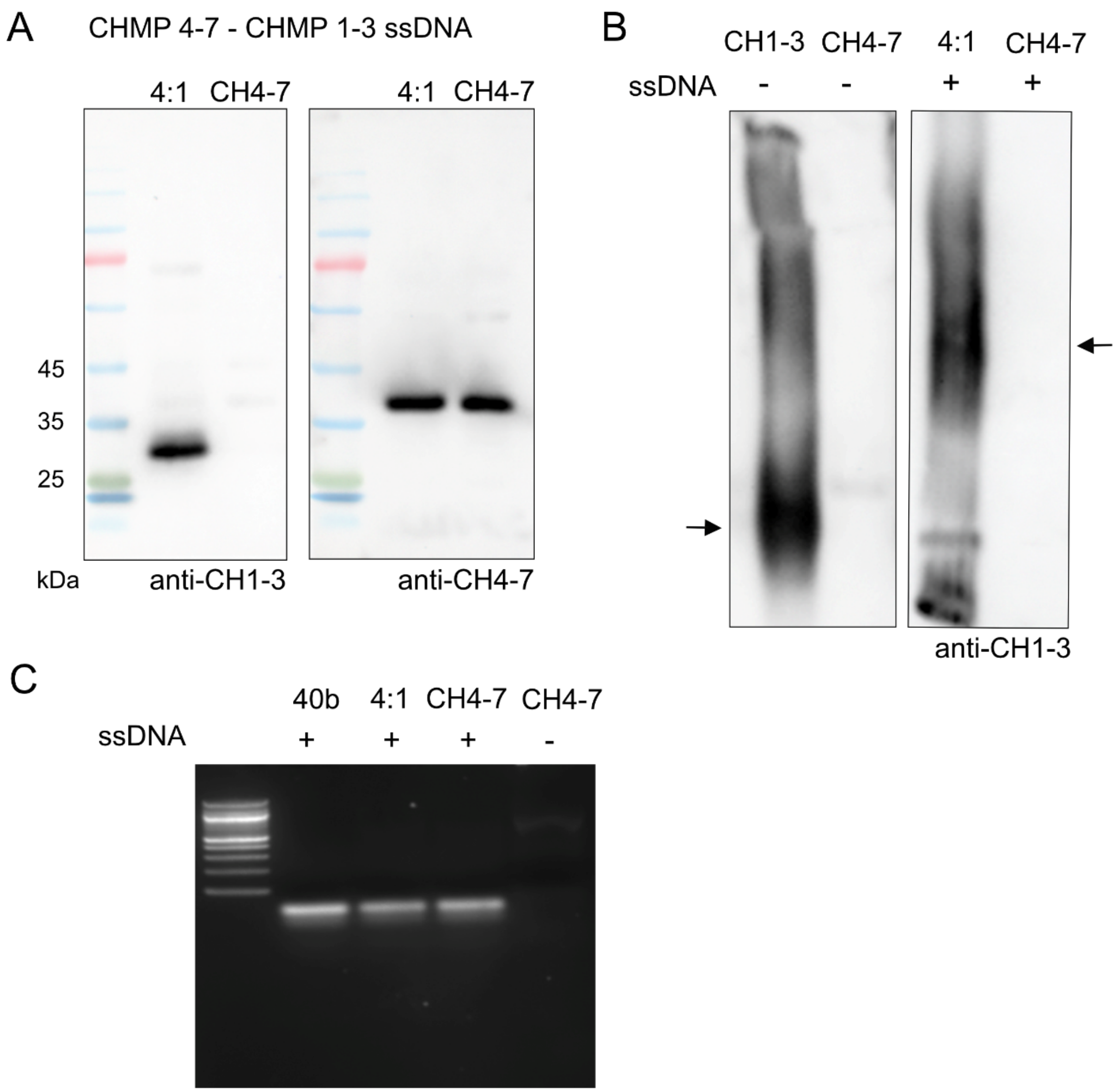

Appendix Figure S6: Biochemical characterization of Loki ESCRT-III filamentous fraction. CHMP4-7 and CHMP1-3 proteins were subjected to polymerization reaction in the presence of ssDNA (10 μM), as described in method section. A sample of filamentous fraction that were mounted on EM grids was loaded on either denaturing SDS-PAGE gel (A), native SDS-PAGE gel (B) or agarose gel (C). Protein gels were then subjected to antibody staining, using custom-made antibodies raised again the purified protein samples. **A.** Western blot analysis showing the presence of both CHMP4-7 and CHMP1-3 in the filament fraction of samples containing CHMP4-7-

CHMP1-3 4:1 molar ratio (4:1) or CHMP4-7 alone in the presence of ssDNA (10 μM). **B.** Native gel showing the typical molecular weight obtained for CHMP1-3 alone and in the presence of CHMP4-7 and ssDNA. The molecular weight shift obtained in the presence of CHMP4-7 and ssDNA (see arrows) strongly supports the association of CHMP1-3 with ESCRT-III filaments. **C.** pure ssDNA probe (left) and samples of fractions that were assembled at the indicated conditions were loaded on agarose gel and stained with ethidium bromide.

Appendix Figure S7

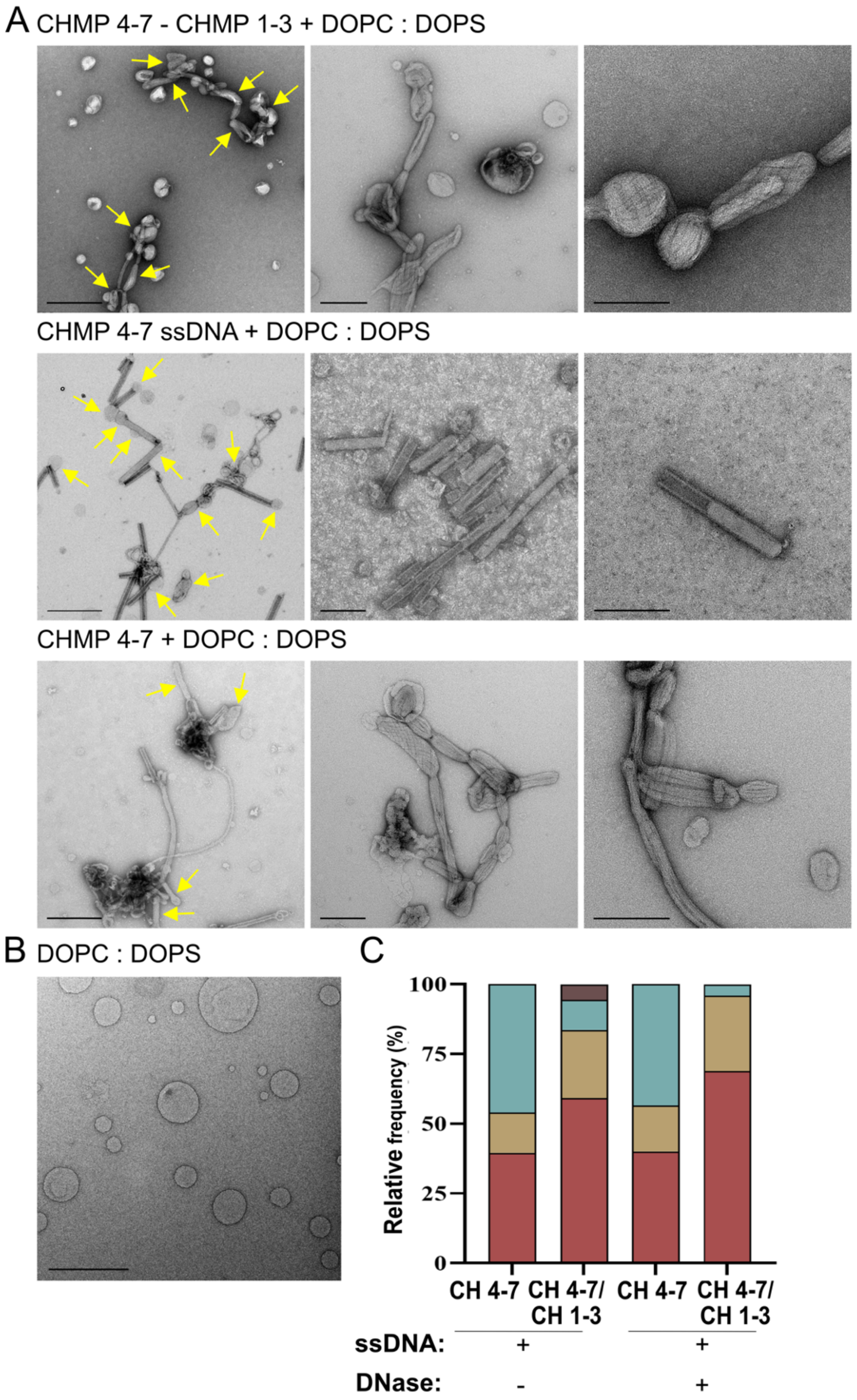

Appendix Figure S7: Interactions of Loki ESCRT-III filaments with negatively charged SUVs (DOPC:DOPS 1:1). A. Representative negative stain TEM images of Loki ESCRT-III filaments that were assembled at the indicated conditions and incubated for 1hour with DOPC:DOPS SUVs. Zoom out images are shown on left panels. Arrows indicate interactions between SUVs and filaments. Corresponds to quantification shown in Fig. 6C. Scale left panels = 500 nm, middle and right panels =200 nm. **B.** A representative negative stain TEM image of DOPC:DOPS SUVs prior to incubation with Loki ESCRT-III. (Scale = 500 nm). **C.** Effect of DNase on pre-assembled ESCRT-III-SUV complexes. ESCRT-III-SUV complexes were generated as in A and were subjected (or not) to DNase treatment and the distribution of the different vesicle-tube interactions observed under the specified conditions was quantified. Note that DNase was not able to disrupt pre-assembled ESCRT-III-SUV interactions (categories are as in Fig. 6C). Data in all panels was reproduced in at least two independent experiments.

Appendix Figure S8

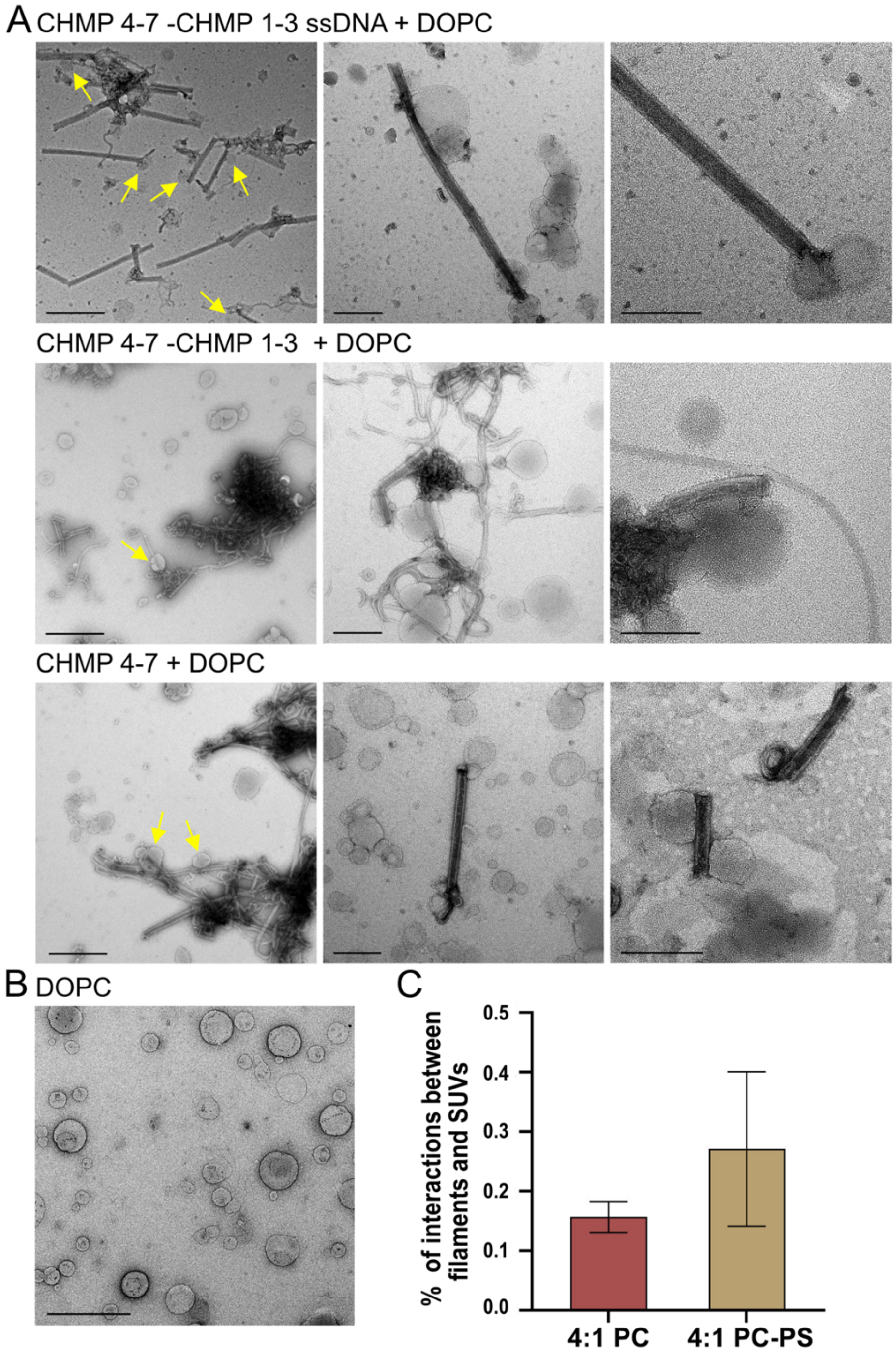

Appendix Figure S8: Interactions of Loki ESCRT-III filaments with DOPC SUVs. A. Representative negative stain TEM images of Loki ESCRT-III filaments that were assembled at the indicated conditions and incubated for 1hour with DOPC SUVs. Zoom out images are shown on left panels. Arrows indicate interactions between SUVs and filaments. Scale: left panels = 500 nm, middle and right panels =200 nm. Note that the most prominent interaction is adhesion of the vesicle to the outside of the tube. **B.** representative negative stain TEM image of DOPC SUVs prior to incubation with Loki ESCRT-III. (Scale = 500 nm). **C.** Percentages of interactions between Loki CHMP 4-7 – CHMP 1-3 (4:1) filaments with SUVs comprised of DOPC or DOPC-DOPS (1:1), showing preference to DOPS containing vesicles. Data in all panels was reproduced in at least two independent experiments.

REFERENCES

Di Tommaso P, Moretti S, Xenarios I, Orobitg M, Montanyola A, Chang JM, Taly JF, Notredame C (2011) T-Coffee: a web server for the multiple sequence alignment of protein and RNA sequences using structural information and homology extension. *Nucleic Acids Res* 39: W13-17

Nachmias D, Melnikov N, Zorea A, Sharon M, Yemini R, De-Picchoto Y, Tsirkas I, Aharoni A, Frohn B, Schwille P *et al* (2022) Asgard ESCRT-III and VPS4 reveal conserved chromatin binding properties of the ESCRT machinery. *ISME J*

Waterhouse AM, Procter JB, Martin DM, Clamp M, Barton GJ (2009) Jalview Version 2--a multiple sequence alignment editor and analysis workbench. *Bioinformatics* 25: 1189-1191
