# Supplementary material for: The Asgard archaeal ESCRT-III system forms helical filaments and remodels eukaryotic-like membranes
Source: EMBO J. 2025 Jan 3;44(3):665–81. doi: 10.1038/s44318-024-00346-4 (PMC11791191; doi:10.1038/s44318-024-00346-4)
Supplement: Supplementary file 3 — Movie EV1 [file 44318_2024_346_MOESM3_ESM.zip › Movie EV1 legend.docx]

Movie EV1

a representative reconstructed tomogram of cryo-fixed filaments-SUV complexes. Samples were prepared using CHMP4-7-CHMP1-3 helical tubes (molar ratio, 4:1) assembled in the presence of ssDNA (10 µM), and incubated with SUVs (DOPC:DOPS, 1:1 ratio).
