# Supplementary figures and images for: The Asgard archaeal ESCRT-III system forms helical filaments and remodels eukaryotic-like membranes
Source: EMBO J. 2025 Jan 3;44(3):665–81. doi: 10.1038/s44318-024-00346-4 (PMC11791191; doi:10.1038/s44318-024-00346-4)

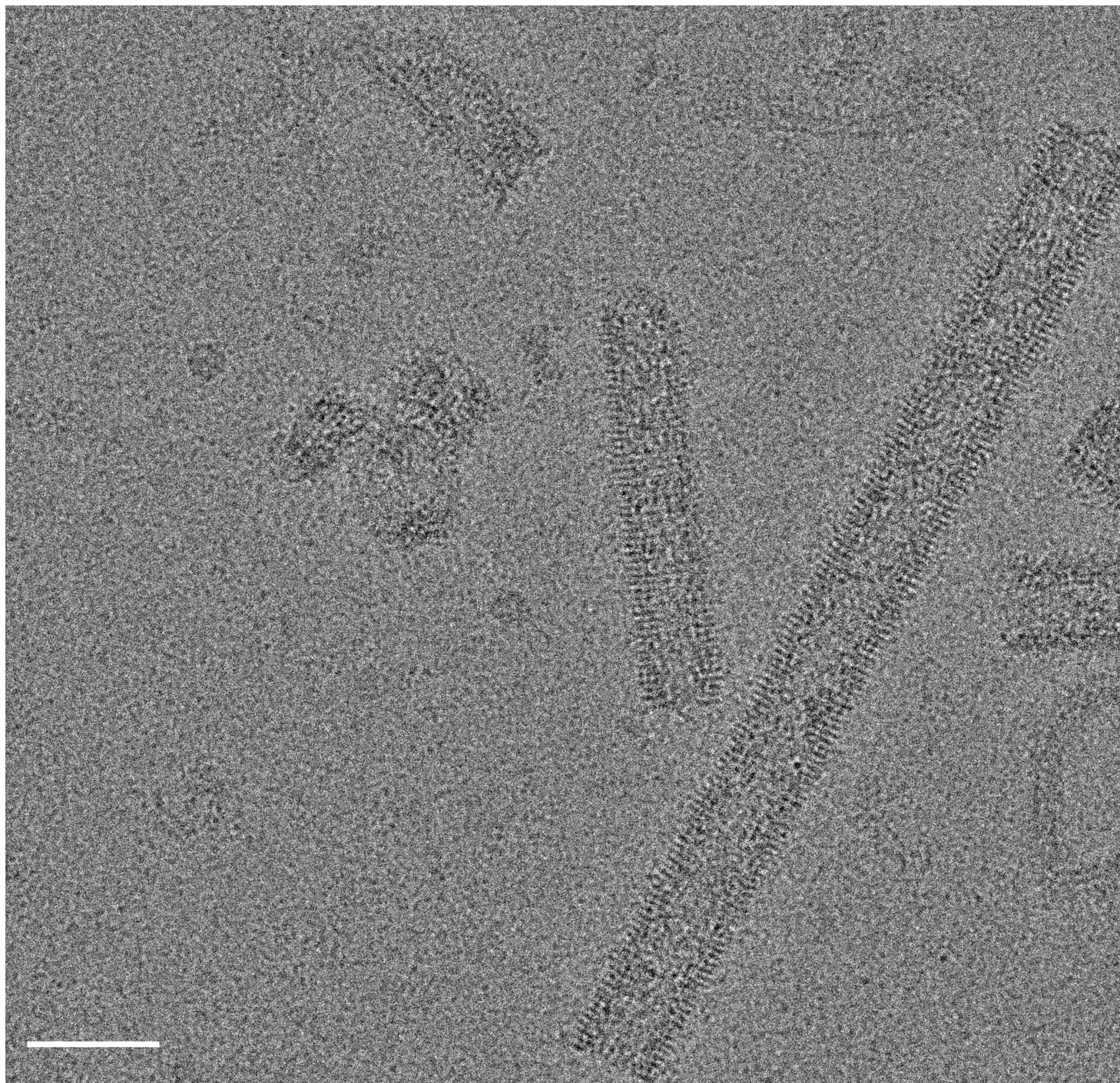

Supplement: Supplementary file 6 — Source data Fig. 1 [file 44318_2024_346_MOESM6_ESM.zip › Fig1/18189201922055671342_CHMP_060921_54_0000_Jun09_21.23.48_rigid_aligned-1.pdf]

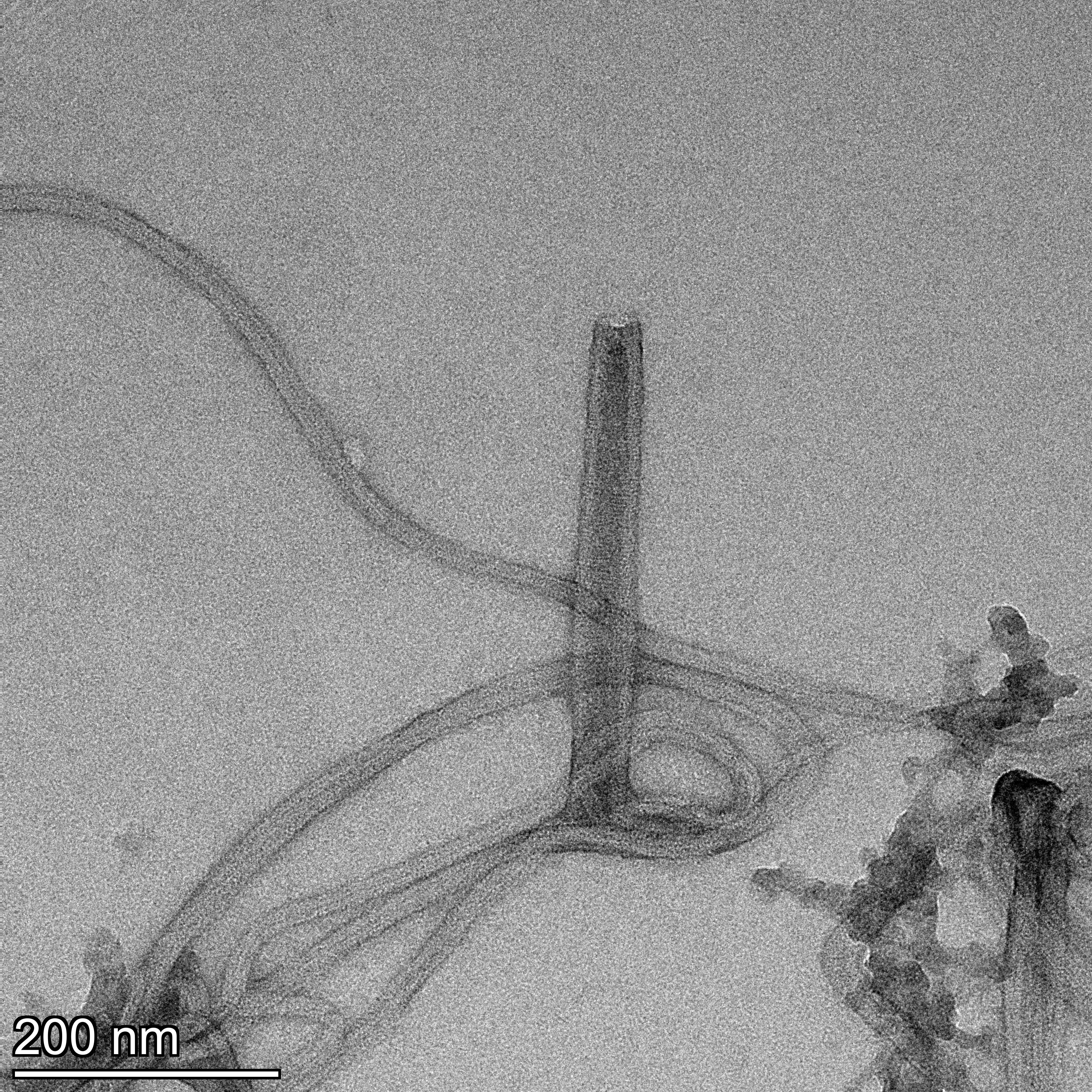

Supplement: Supplementary file 6 — Source data Fig. 1 [file 44318_2024_346_MOESM6_ESM.zip › Fig1/B middle.jpg]

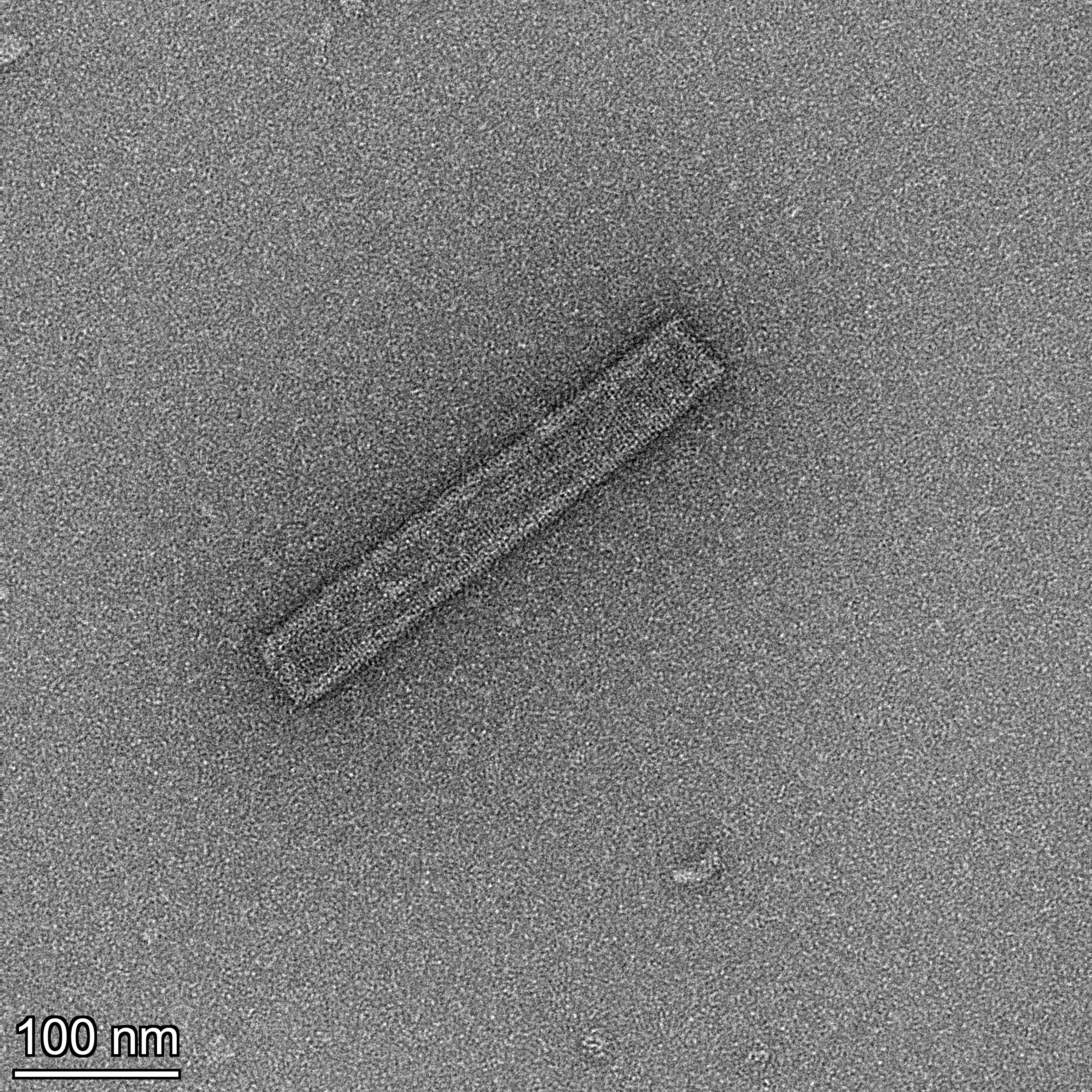

Supplement: Supplementary file 6 — Source data Fig. 1 [file 44318_2024_346_MOESM6_ESM.zip › Fig1/B right.jpg]

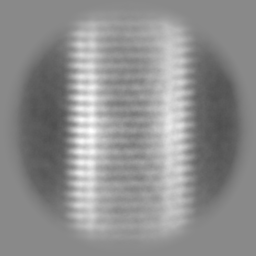

Supplement: Supplementary file 6 — Source data Fig. 1 [file 44318_2024_346_MOESM6_ESM.zip › Fig1/D_image 7_cryosparc_P18_J276_020_class_averages.tif]

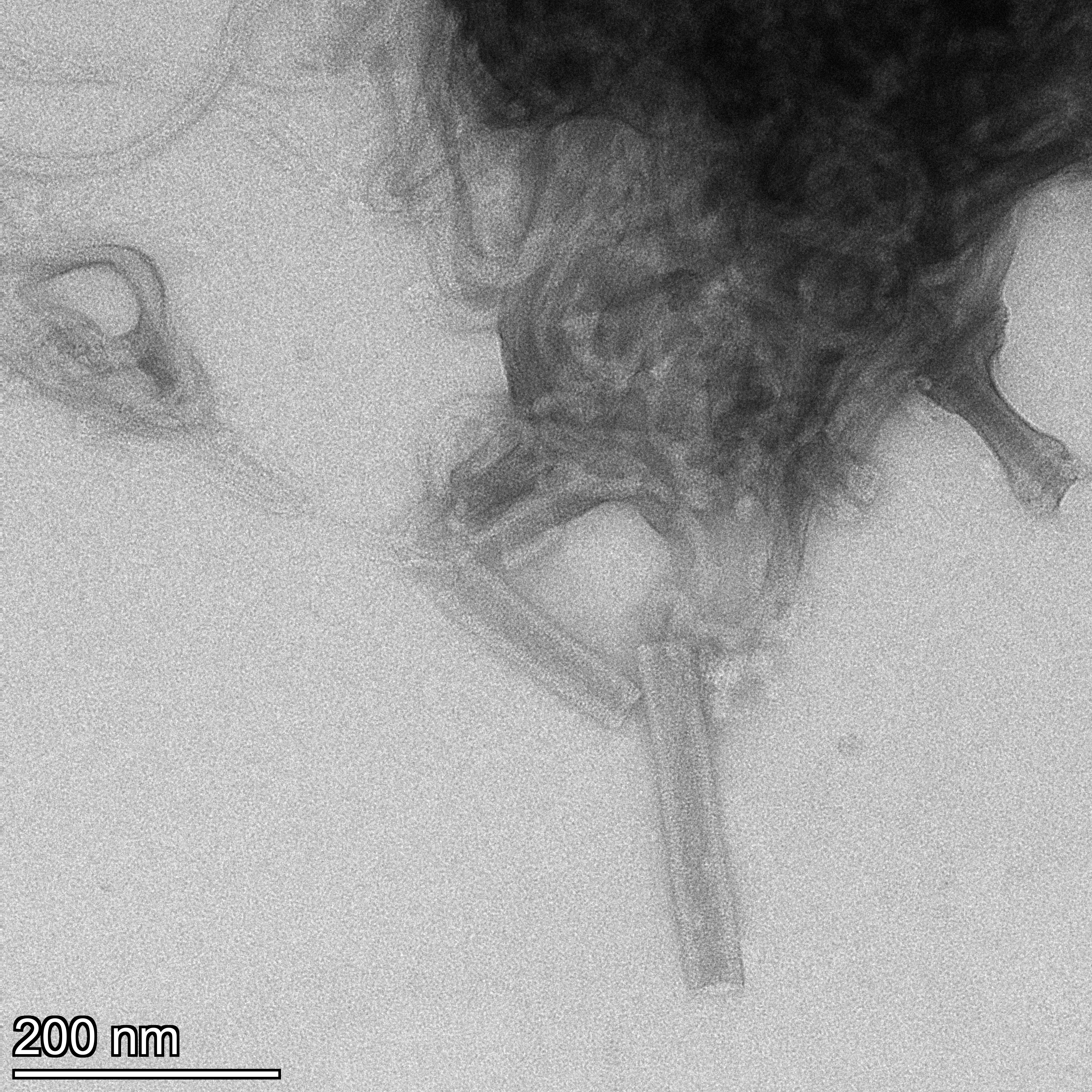

Supplement: Supplementary file 7 — Source data Fig. 2 [file 44318_2024_346_MOESM7_ESM.zip › Fig2/2A top right_c47 73000.jpg]

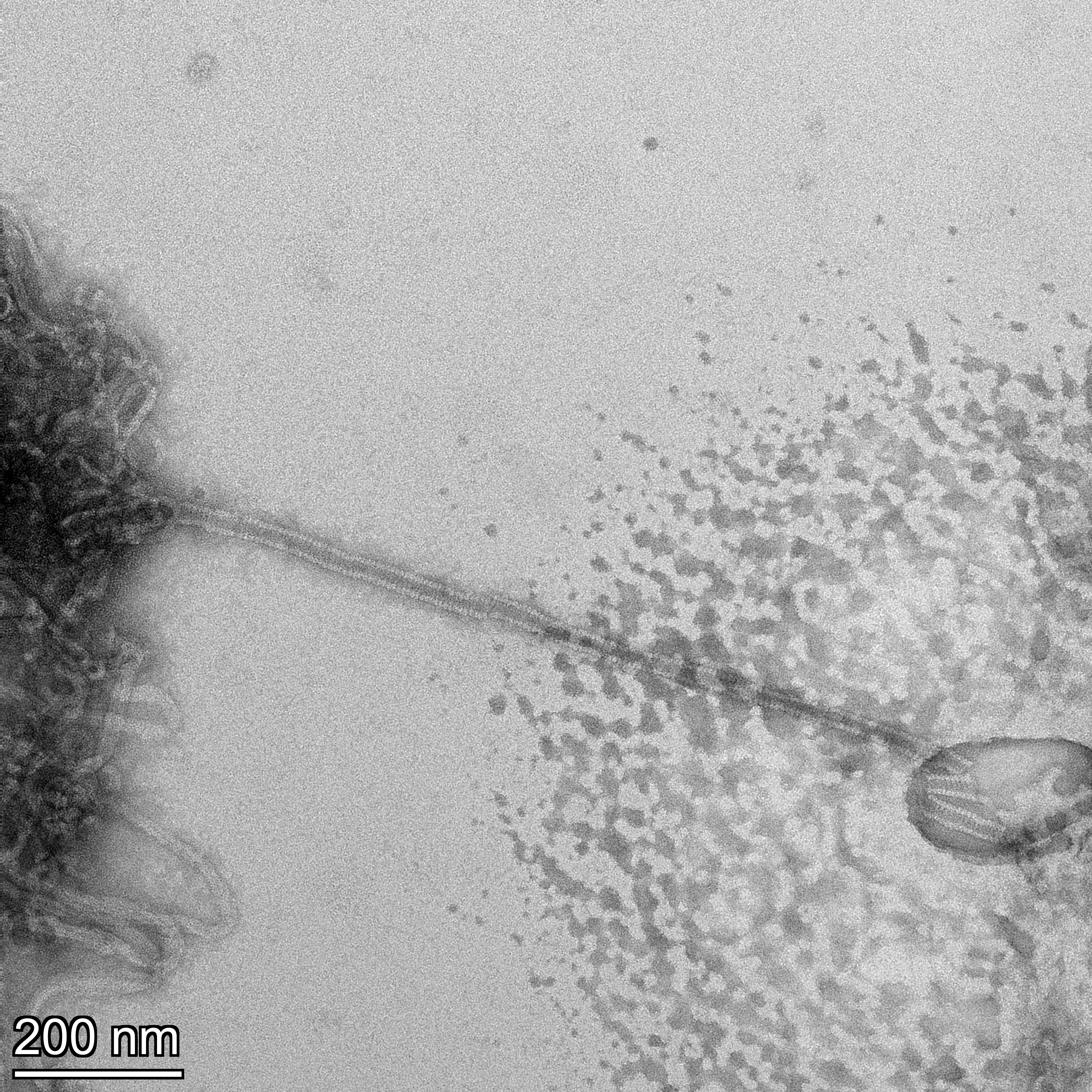

Supplement: Supplementary file 7 — Source data Fig. 2 [file 44318_2024_346_MOESM7_ESM.zip › Fig2/2A bottom right_4-1 45000 x.jpg]

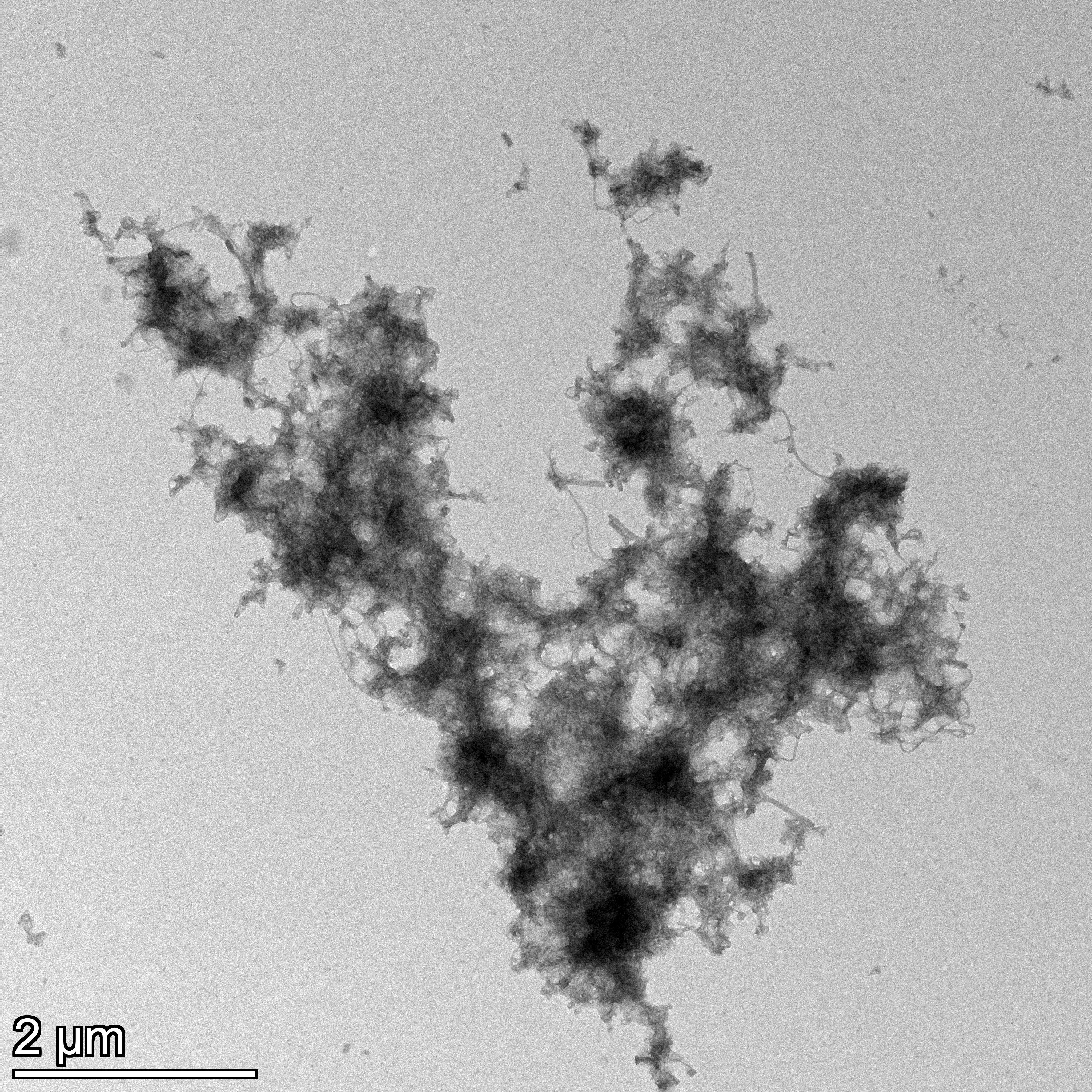

Supplement: Supplementary file 7 — Source data Fig. 2 [file 44318_2024_346_MOESM7_ESM.zip › Fig2/2A bottom left_4-1 6700 x.jpg]

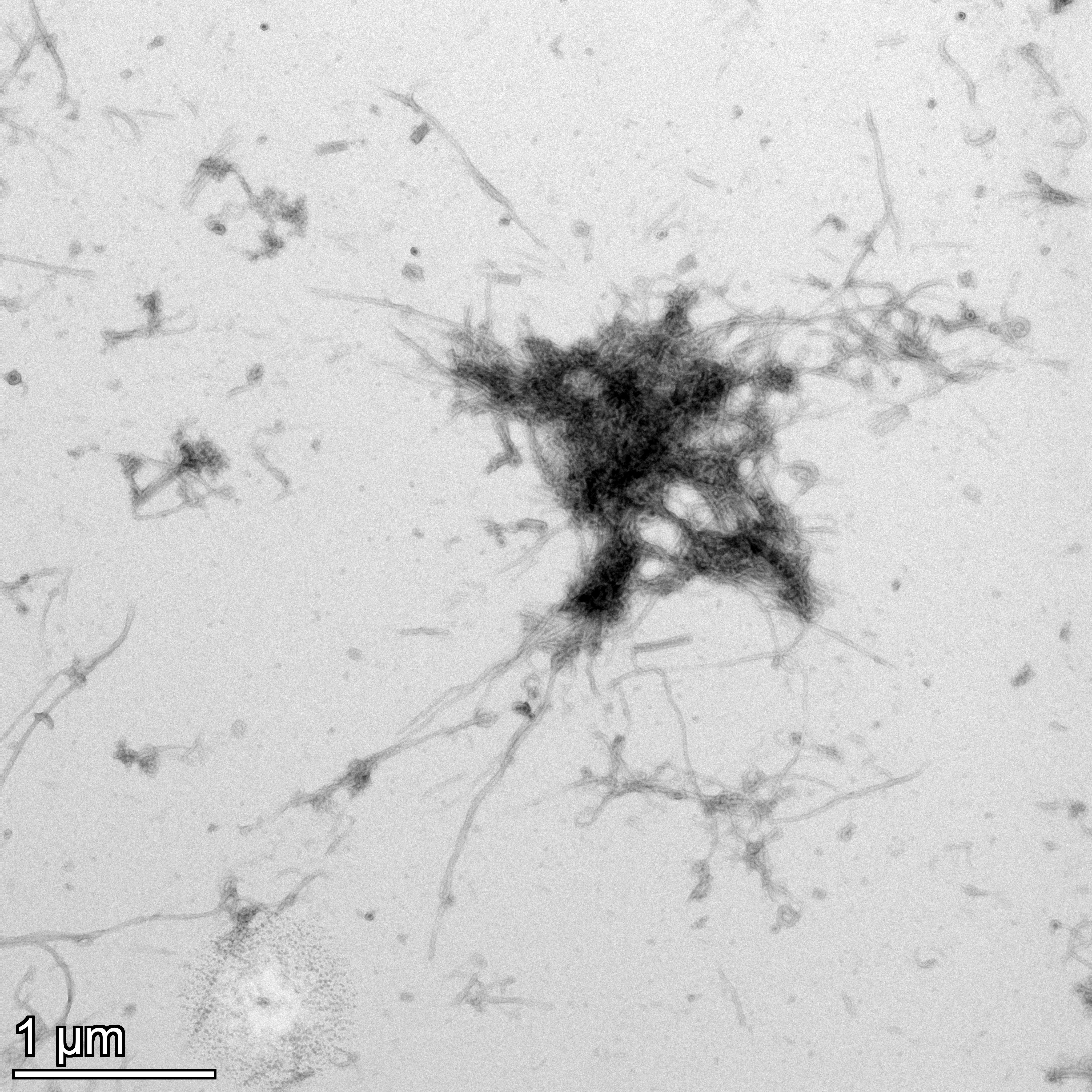

Supplement: Supplementary file 7 — Source data Fig. 2 [file 44318_2024_346_MOESM7_ESM.zip › Fig2/2A top left_C47 11000 x.jpg]

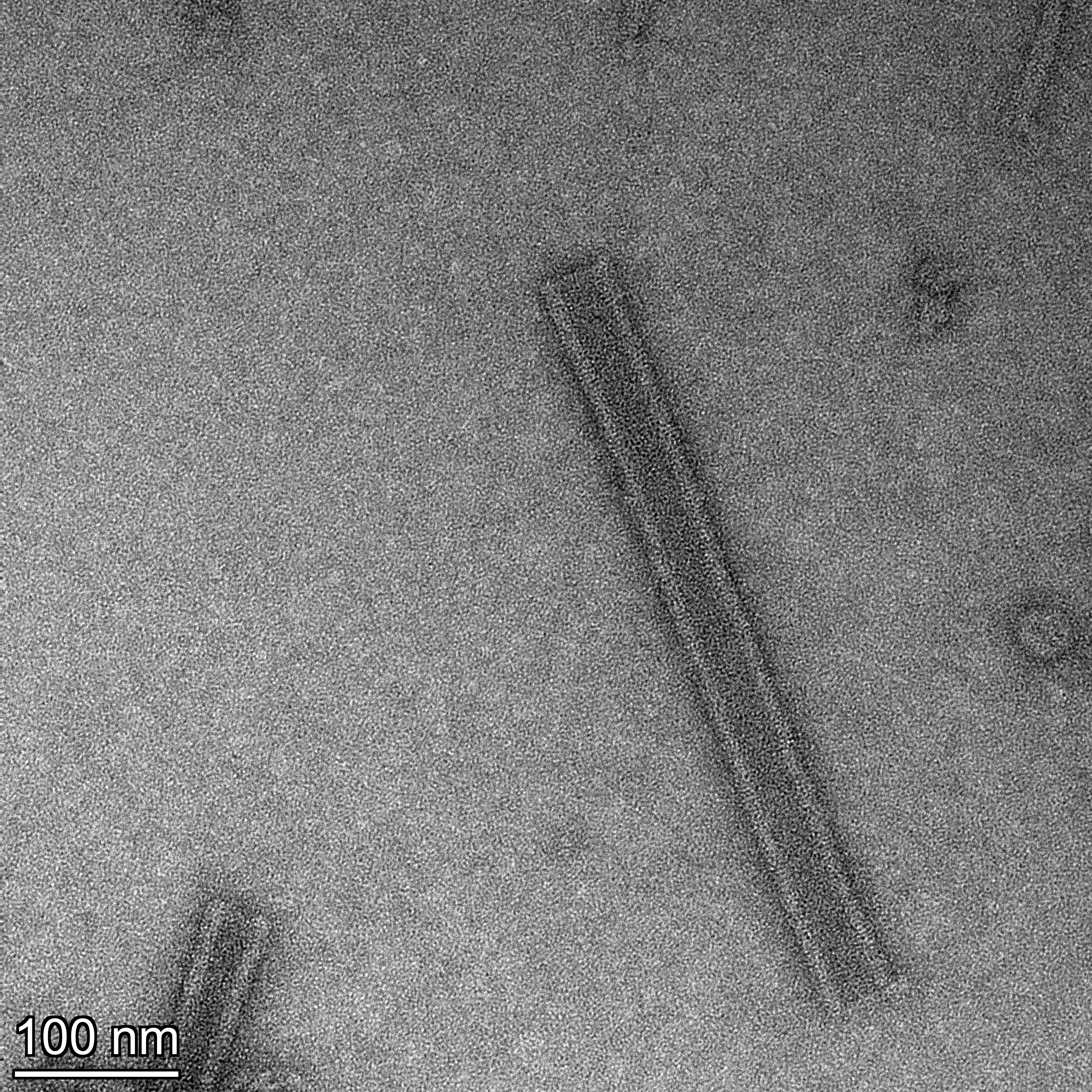

Supplement: Supplementary file 8 — Source data Fig. 3 [file 44318_2024_346_MOESM8_ESM.zip › Fig 3/3A_down rightt.jpg]

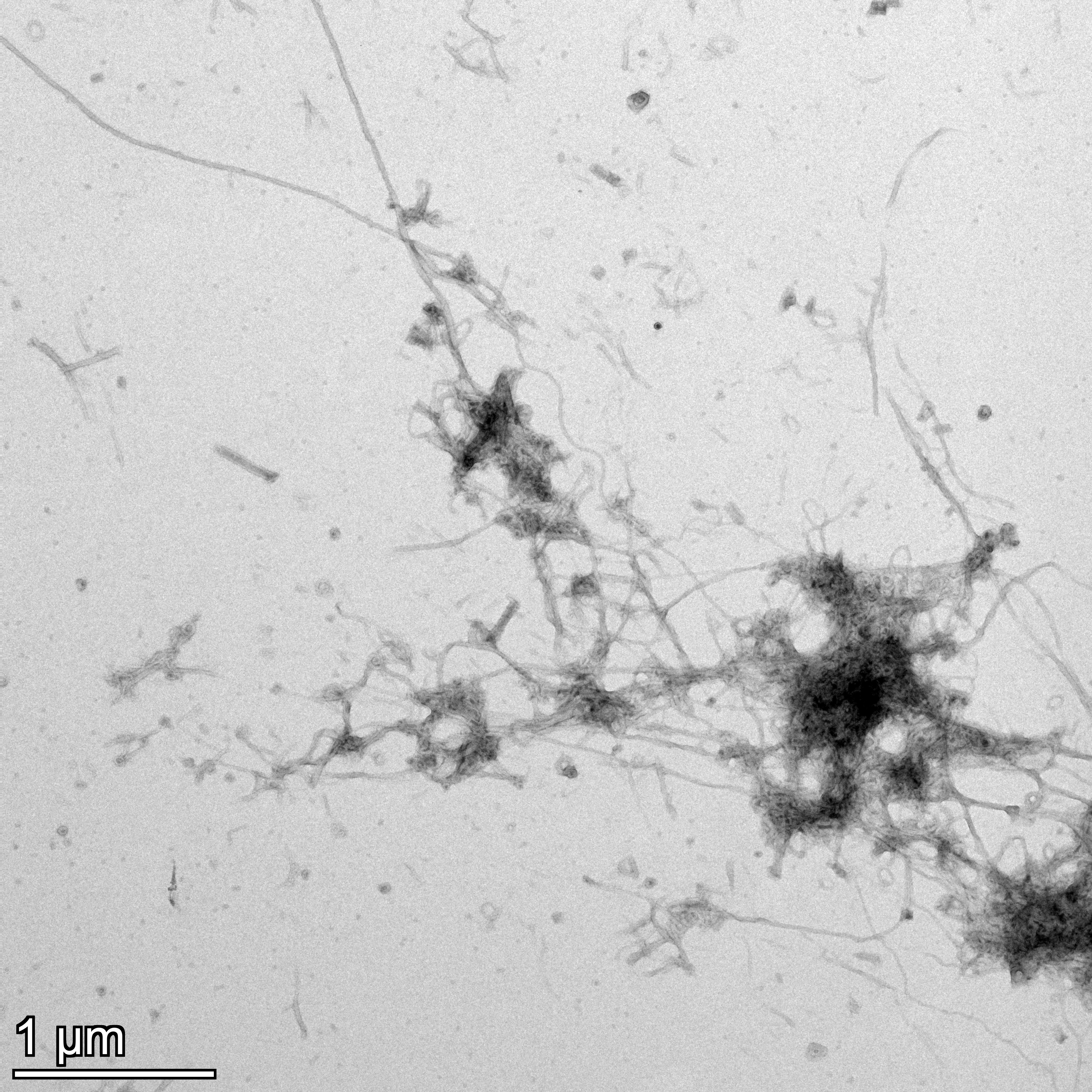

Supplement: Supplementary file 8 — Source data Fig. 3 [file 44318_2024_346_MOESM8_ESM.zip › Fig 3/3B_up left.jpg]

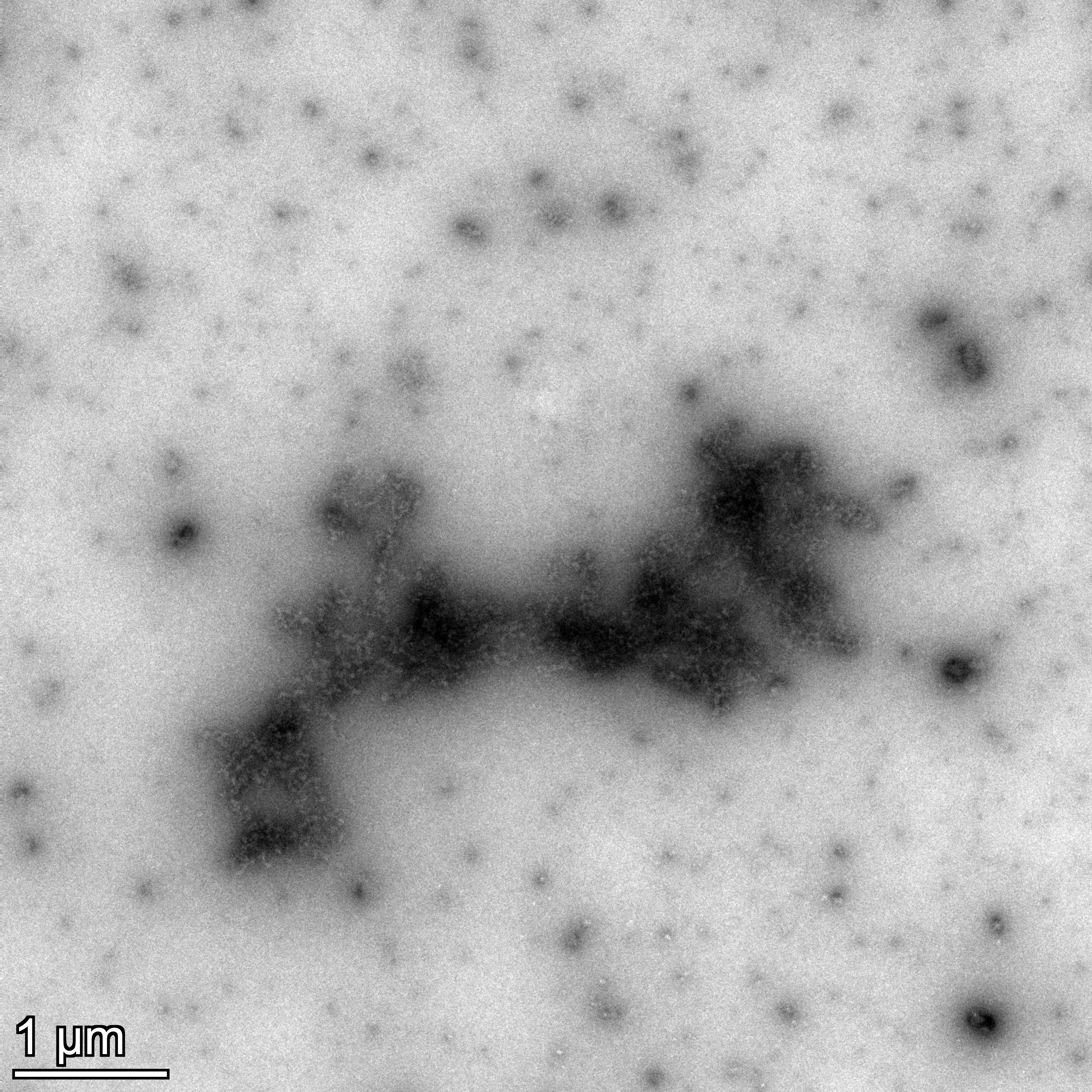

Supplement: Supplementary file 8 — Source data Fig. 3 [file 44318_2024_346_MOESM8_ESM.zip › Fig 3/3E_right.tiff]

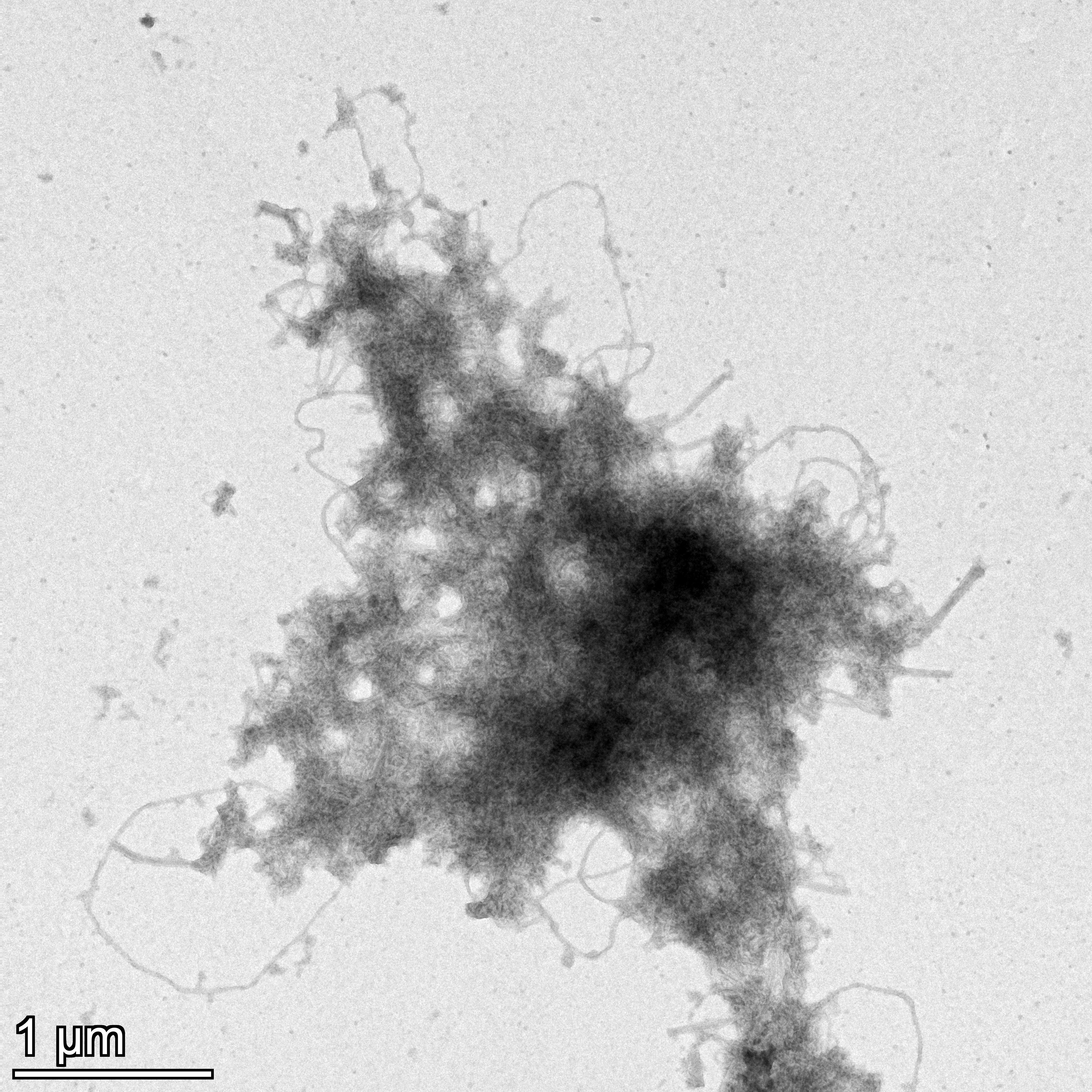

Supplement: Supplementary file 8 — Source data Fig. 3 [file 44318_2024_346_MOESM8_ESM.zip › Fig 3/3A_up left.jpg]

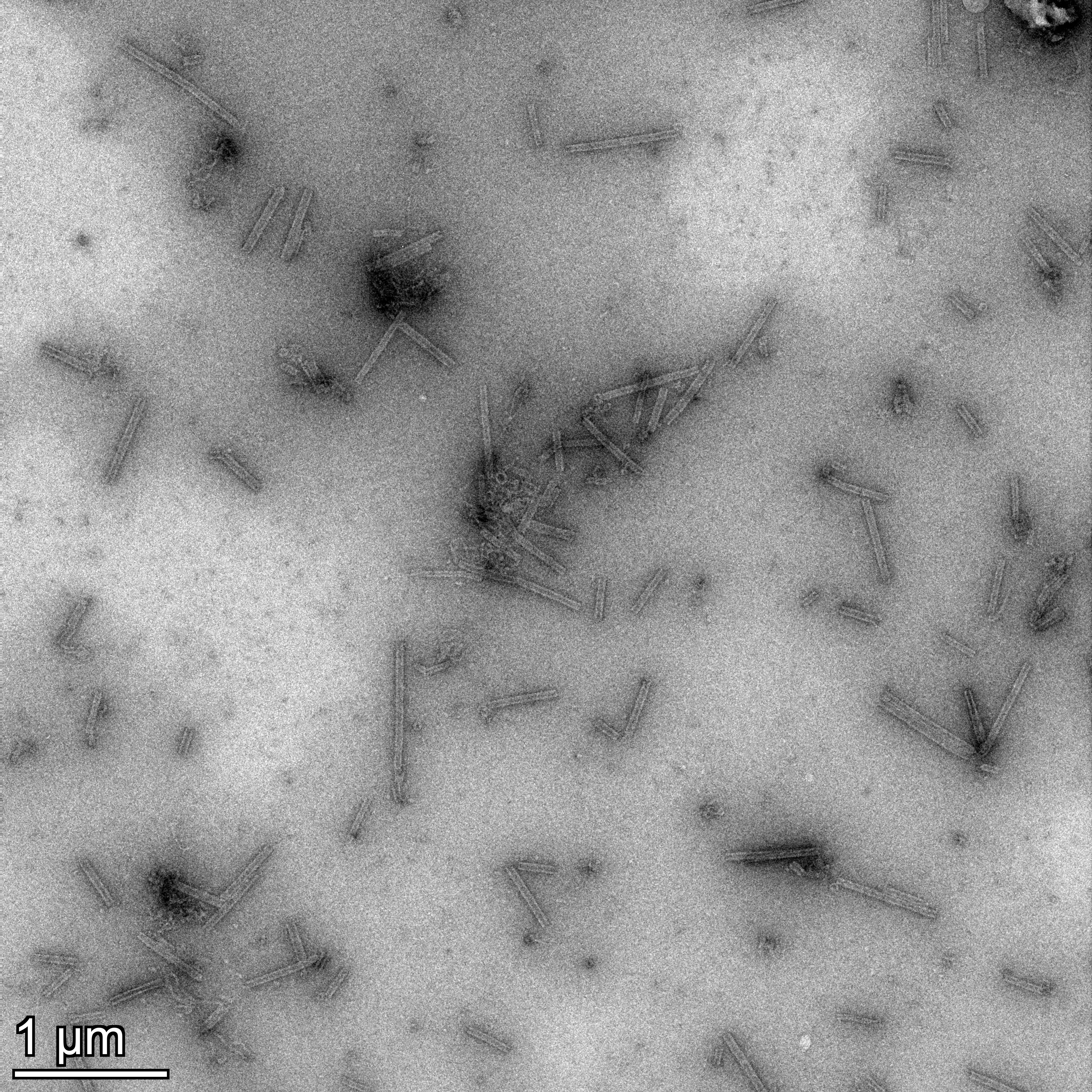

Supplement: Supplementary file 8 — Source data Fig. 3 [file 44318_2024_346_MOESM8_ESM.zip › Fig 3/3A_down left.jpg]

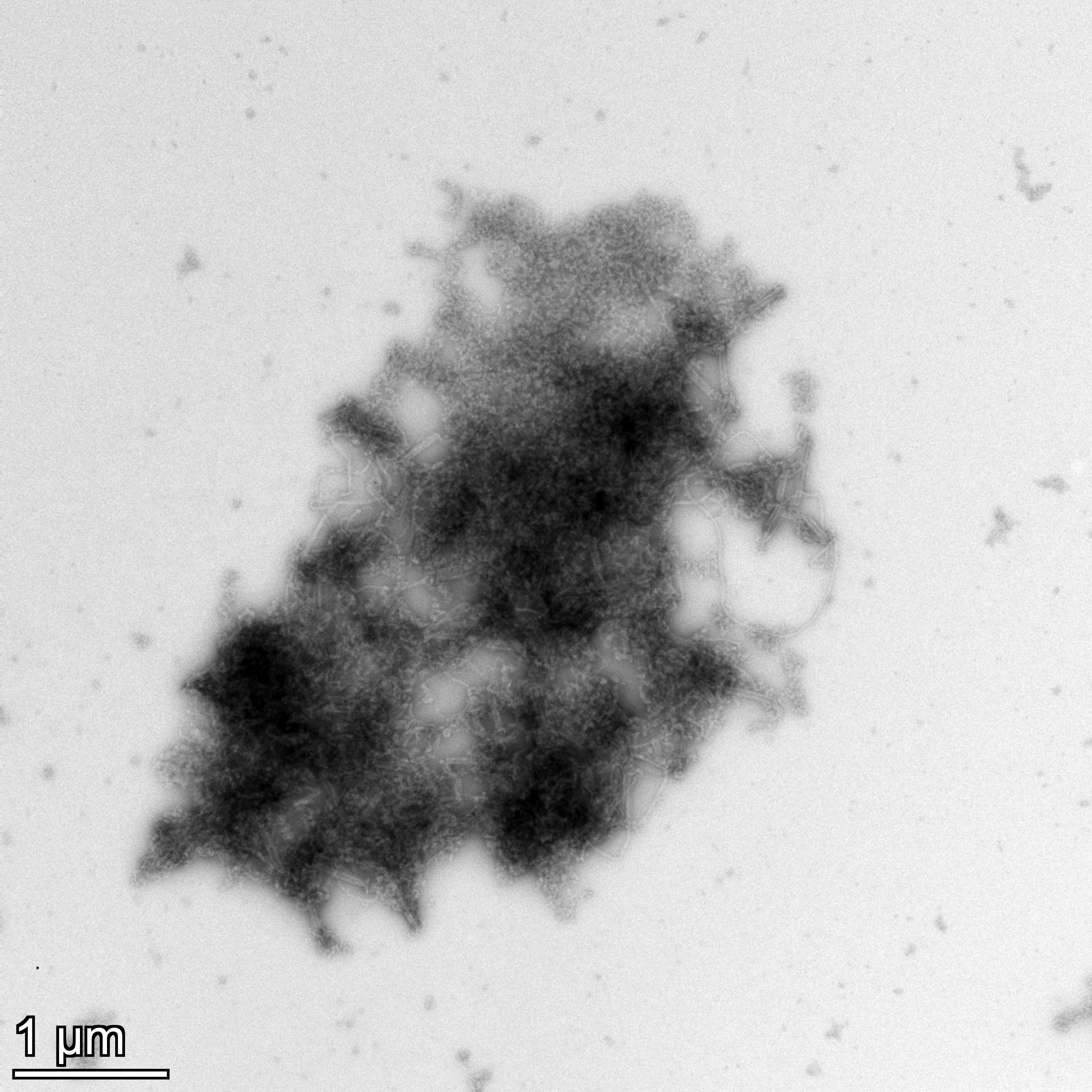

Supplement: Supplementary file 8 — Source data Fig. 3 [file 44318_2024_346_MOESM8_ESM.zip › Fig 3/3E_left.jpg]

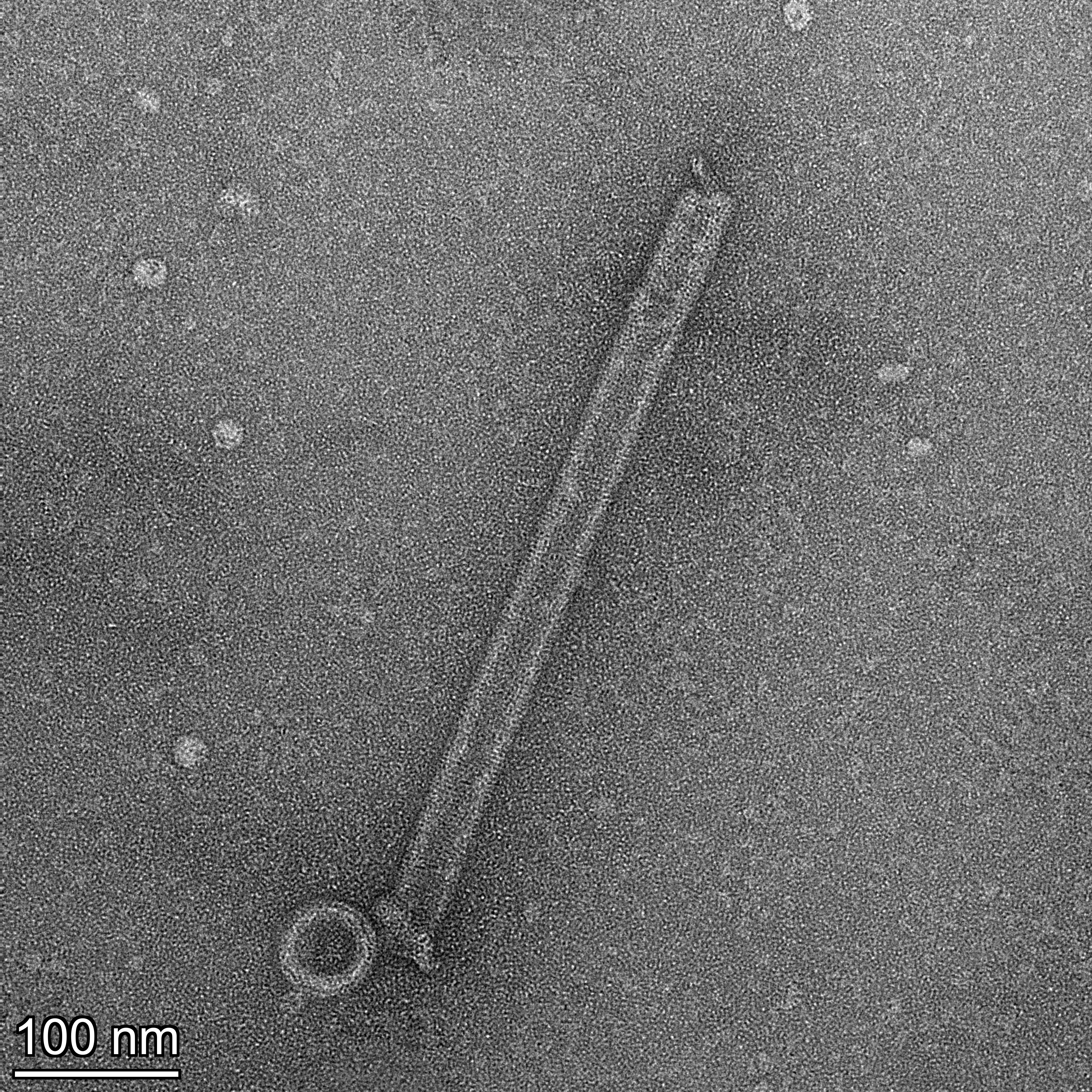

Supplement: Supplementary file 8 — Source data Fig. 3 [file 44318_2024_346_MOESM8_ESM.zip › Fig 3/3B _up right.jpg]

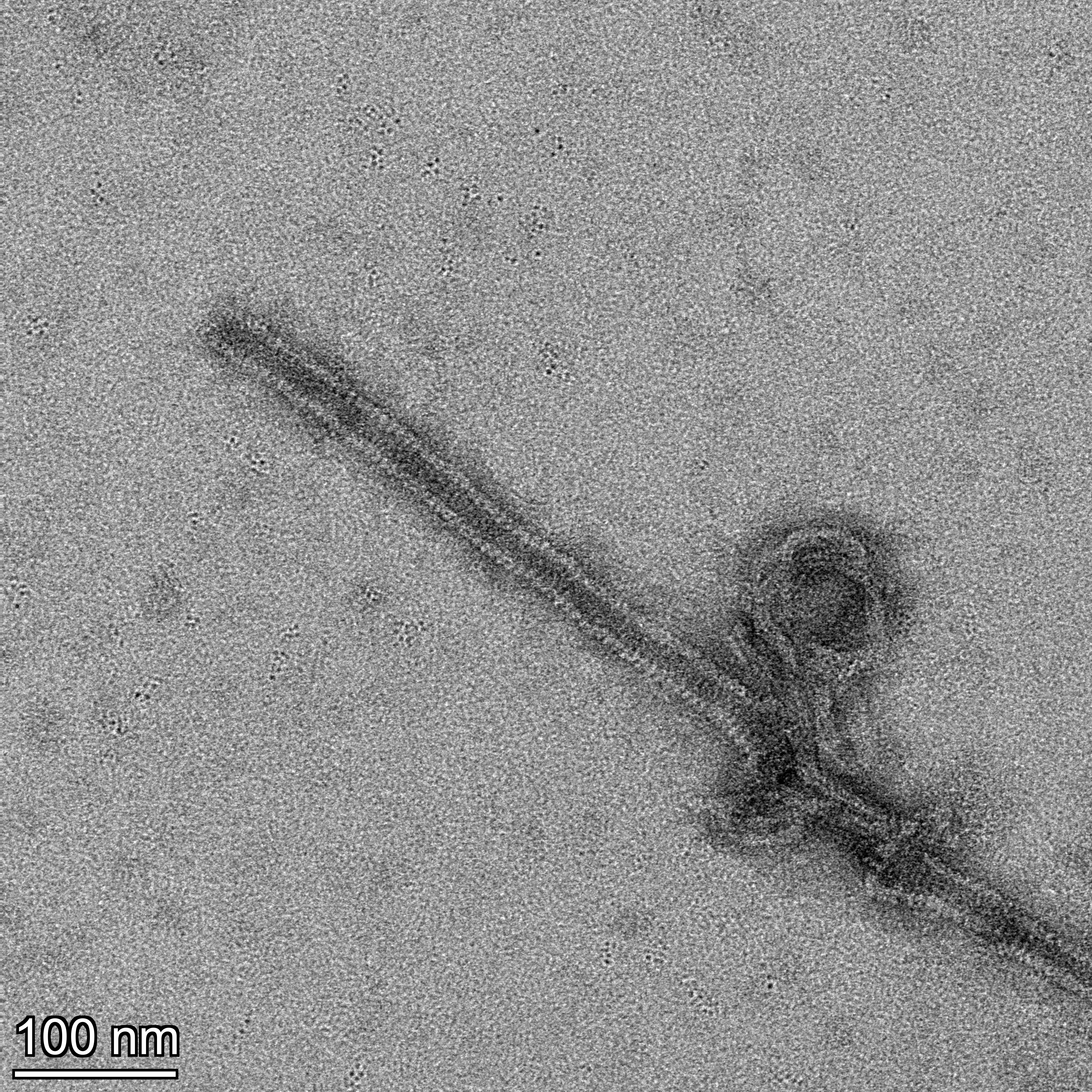

Supplement: Supplementary file 8 — Source data Fig. 3 [file 44318_2024_346_MOESM8_ESM.zip › Fig 3/3A_up right.jpg]

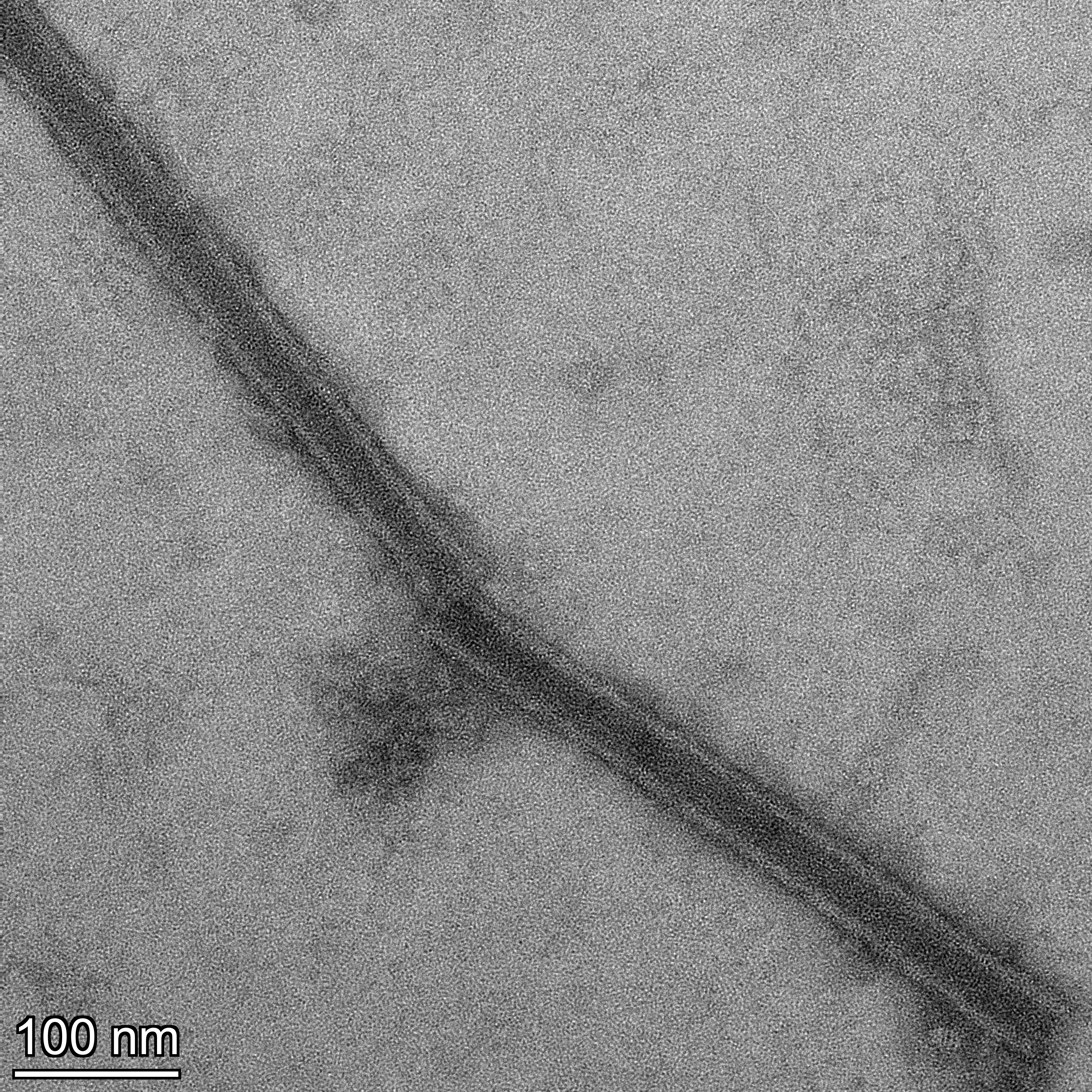

Supplement: Supplementary file 8 — Source data Fig. 3 [file 44318_2024_346_MOESM8_ESM.zip › Fig 3/3B _down right.jpg]

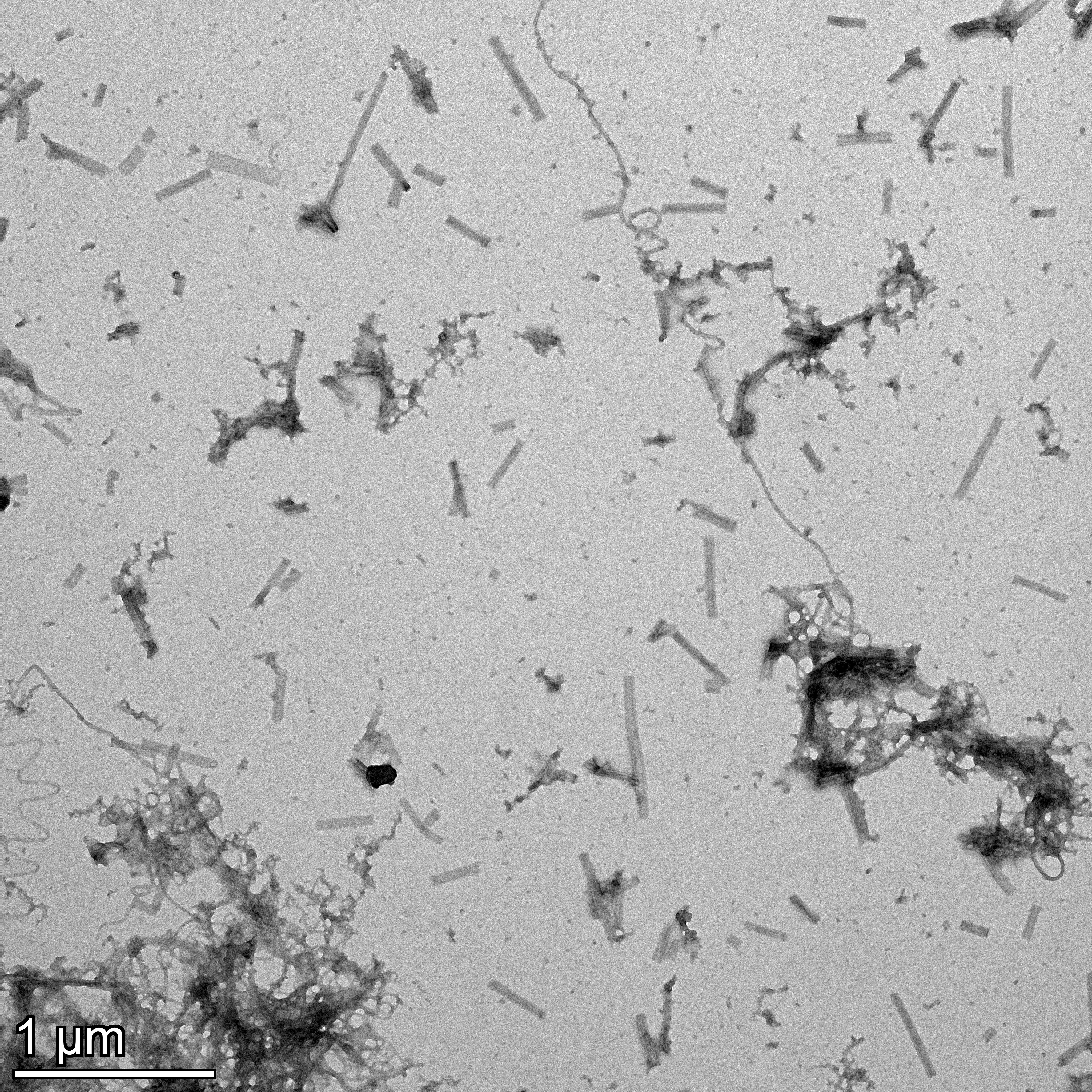

Supplement: Supplementary file 8 — Source data Fig. 3 [file 44318_2024_346_MOESM8_ESM.zip › Fig 3/3B_down left.jpg]

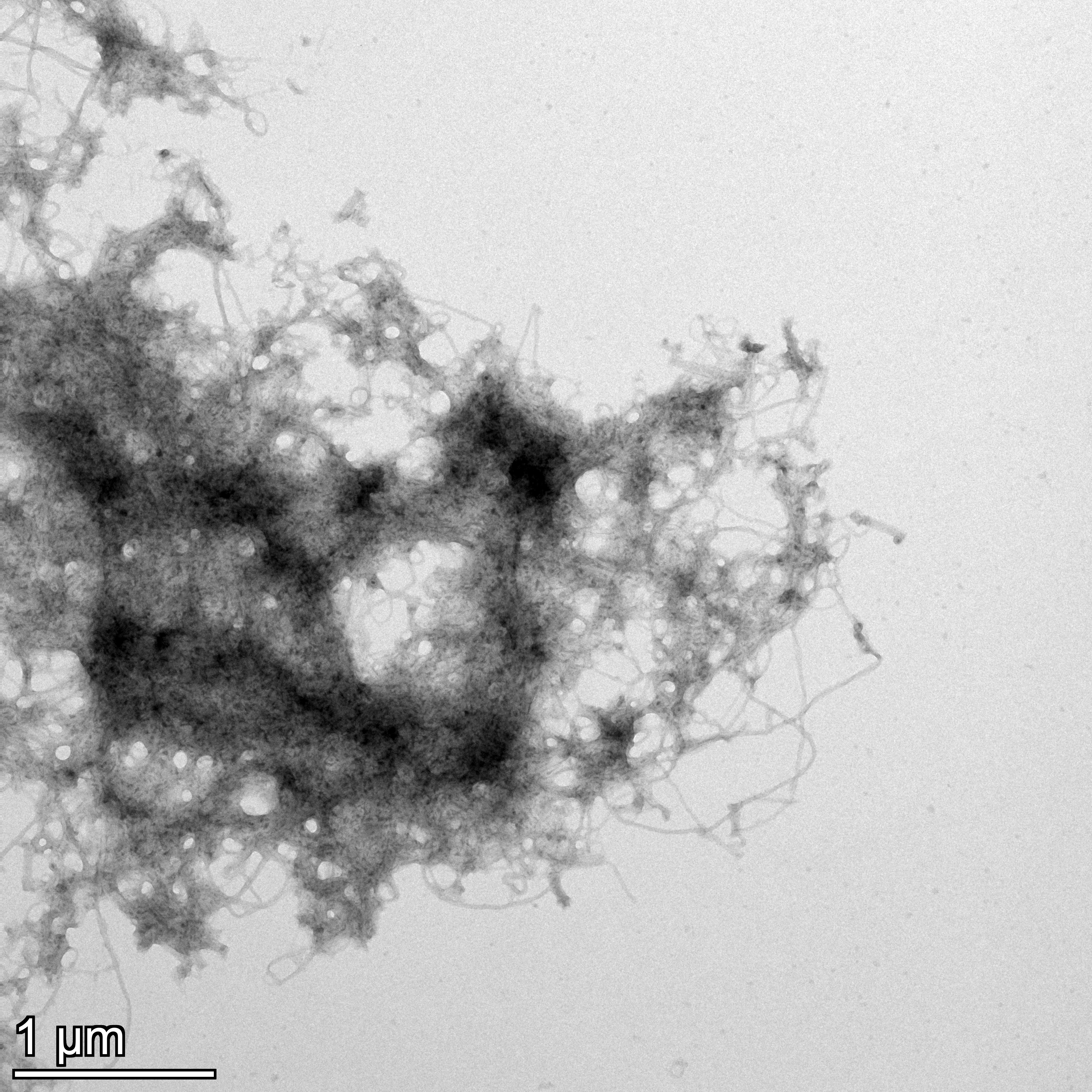

Supplement: Supplementary file 8 — Source data Fig. 3 [file 44318_2024_346_MOESM8_ESM.zip › Fig 3/3E_3.jpg]

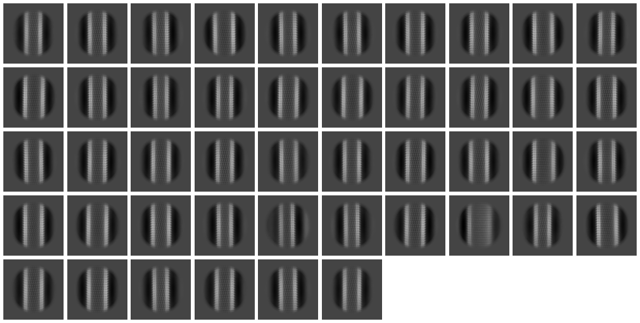

Supplement: Supplementary file 9 — Source data Fig. 4 [file 44318_2024_346_MOESM9_ESM.zip › Fig 4/4B_J242_selected_46_classes.png]

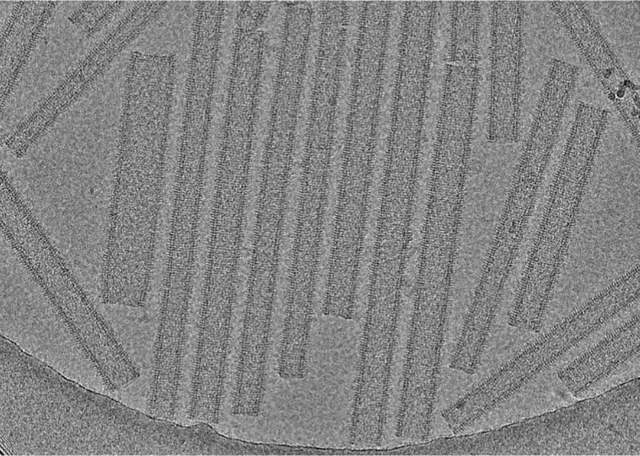

Supplement: Supplementary file 9 — Source data Fig. 4 [file 44318_2024_346_MOESM9_ESM.zip › Fig 4/4A_Micrograph.png]

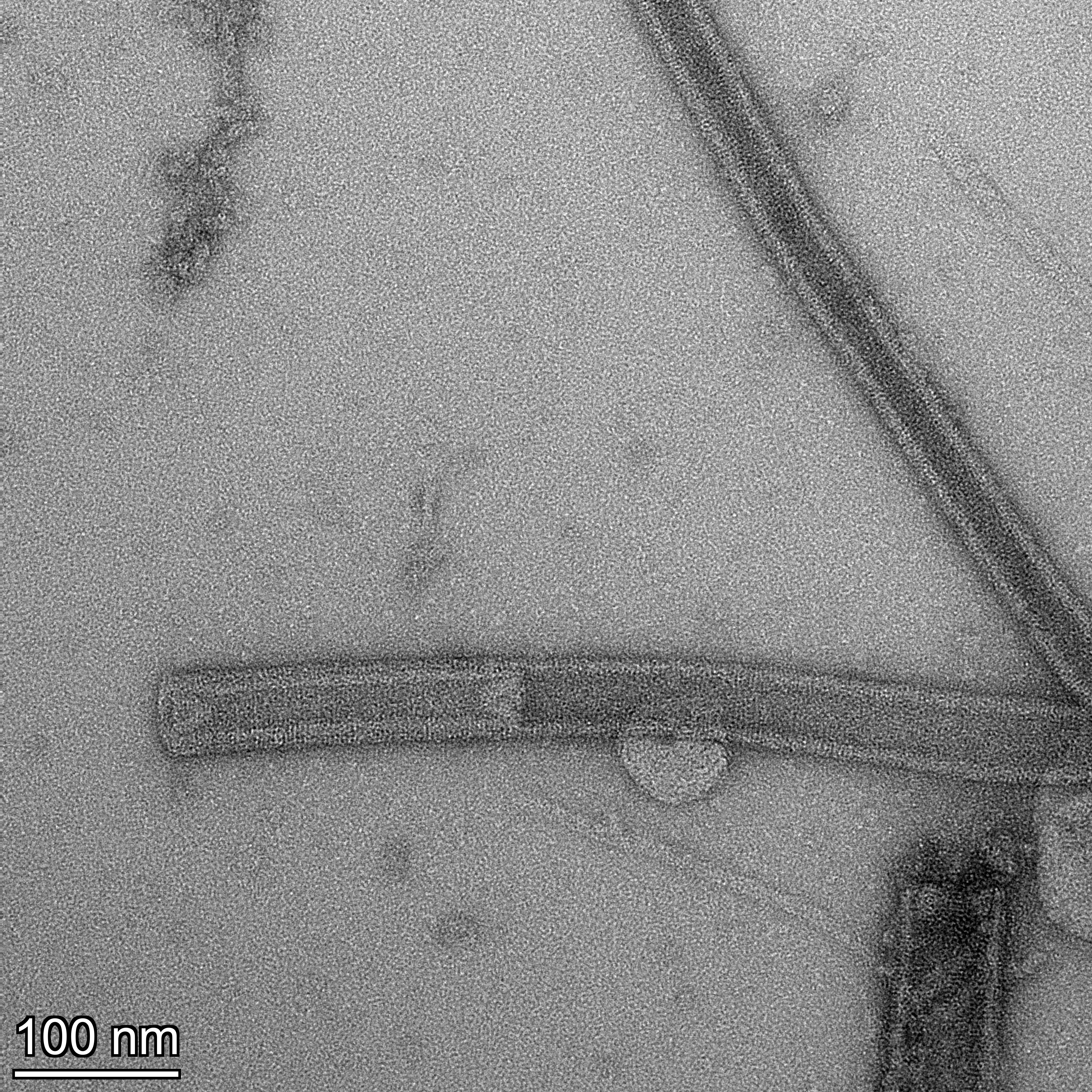

Supplement: Supplementary file 10 — Source data Fig. 6 [file 44318_2024_346_MOESM10_ESM.zip › Fig 6/6C_inside.jpg]

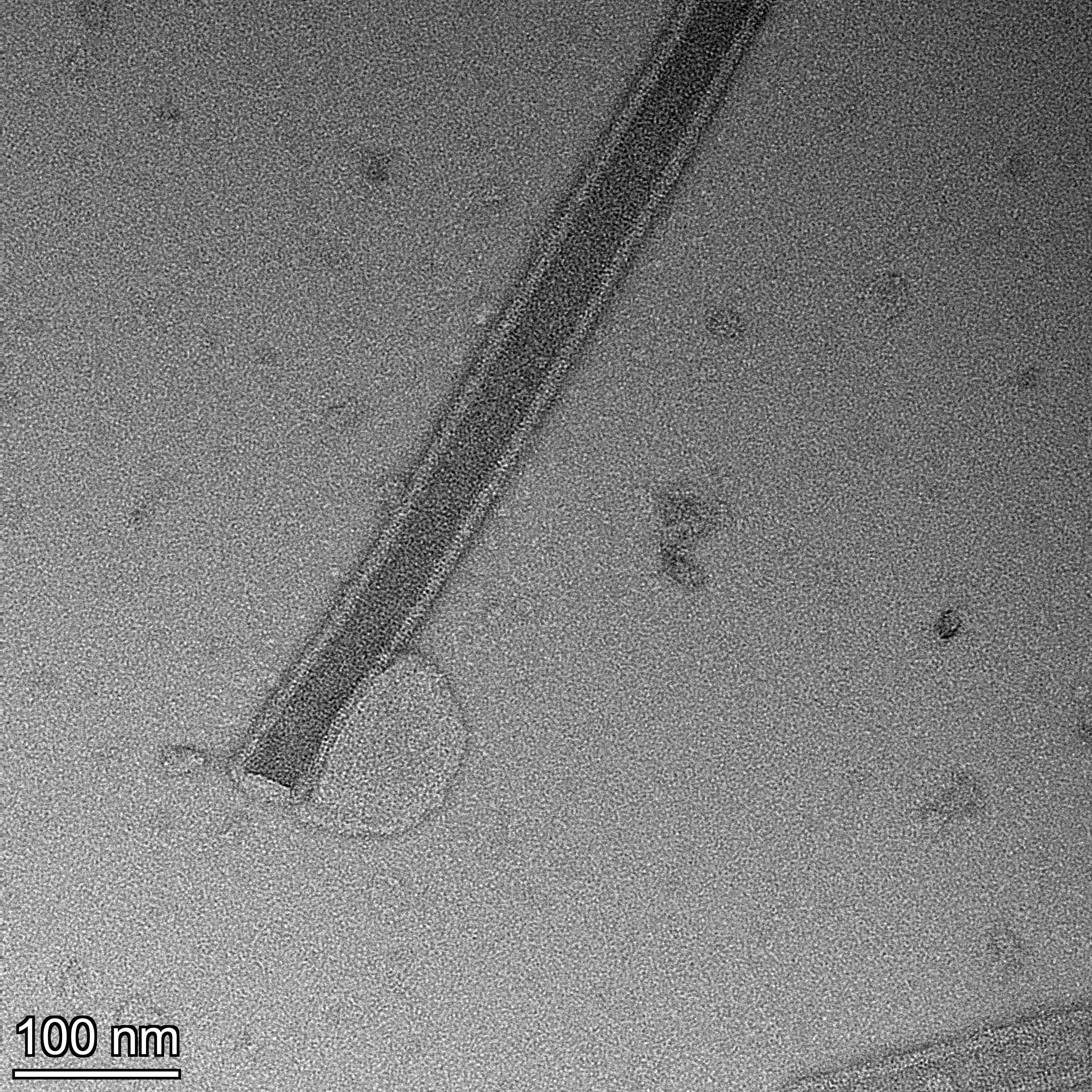

Supplement: Supplementary file 10 — Source data Fig. 6 [file 44318_2024_346_MOESM10_ESM.zip › Fig 6/6C_outside.jpg]

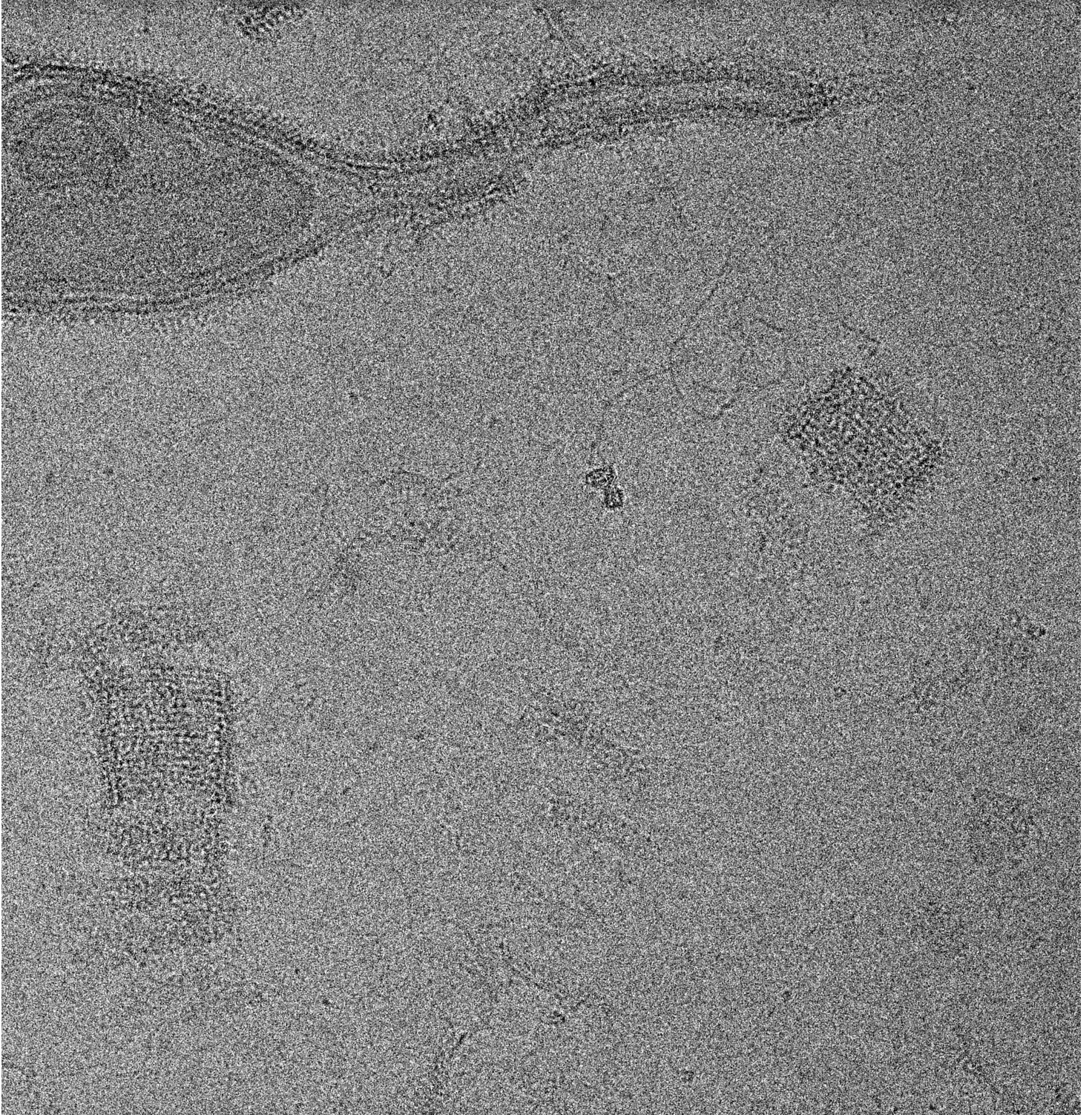

Supplement: Supplementary file 10 — Source data Fig. 6 [file 44318_2024_346_MOESM10_ESM.zip › Fig 6/6B_middle_022222_wDNA_G1_S1_Prev1_scale.pdf]

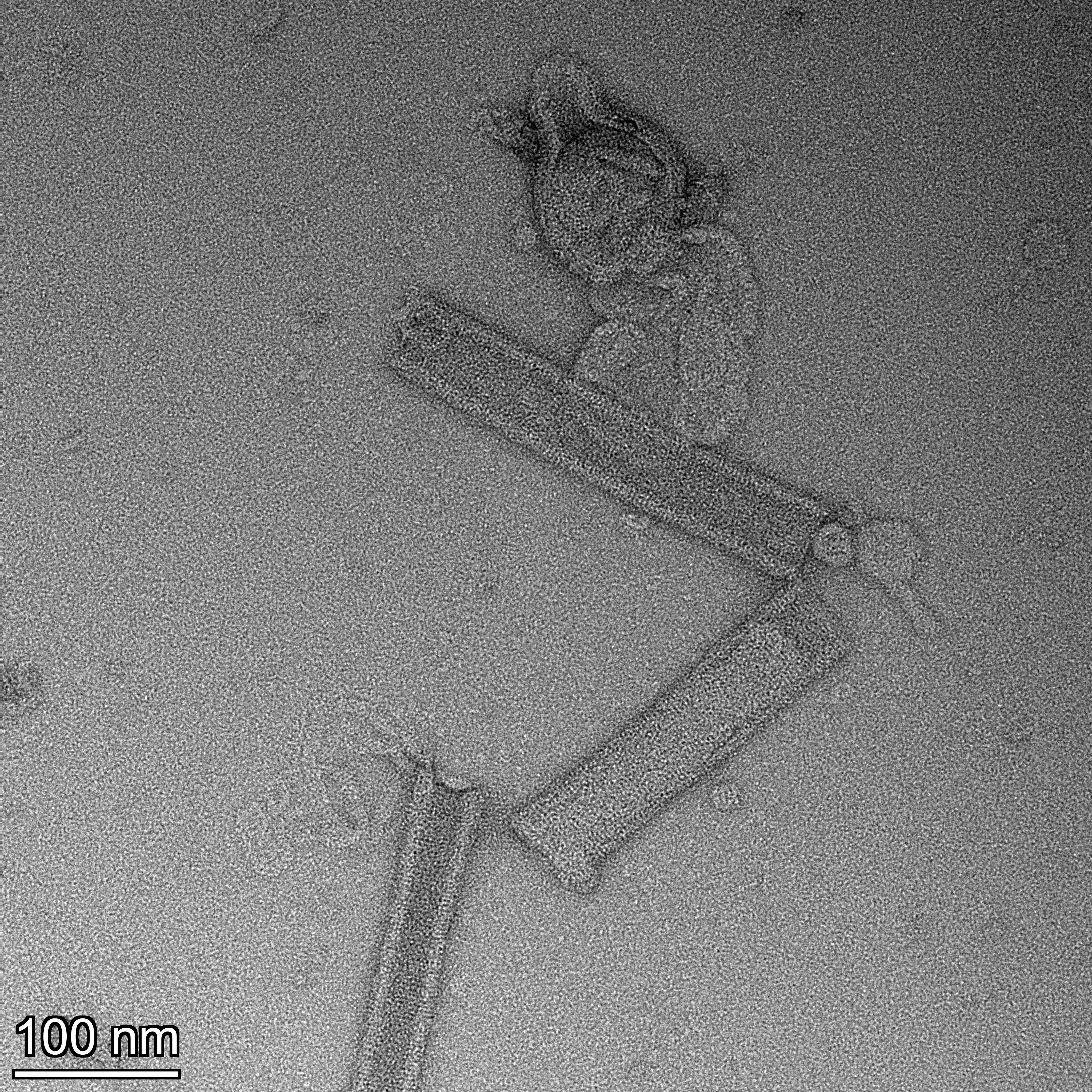

Supplement: Supplementary file 10 — Source data Fig. 6 [file 44318_2024_346_MOESM10_ESM.zip › Fig 6/6A_right.jpg]

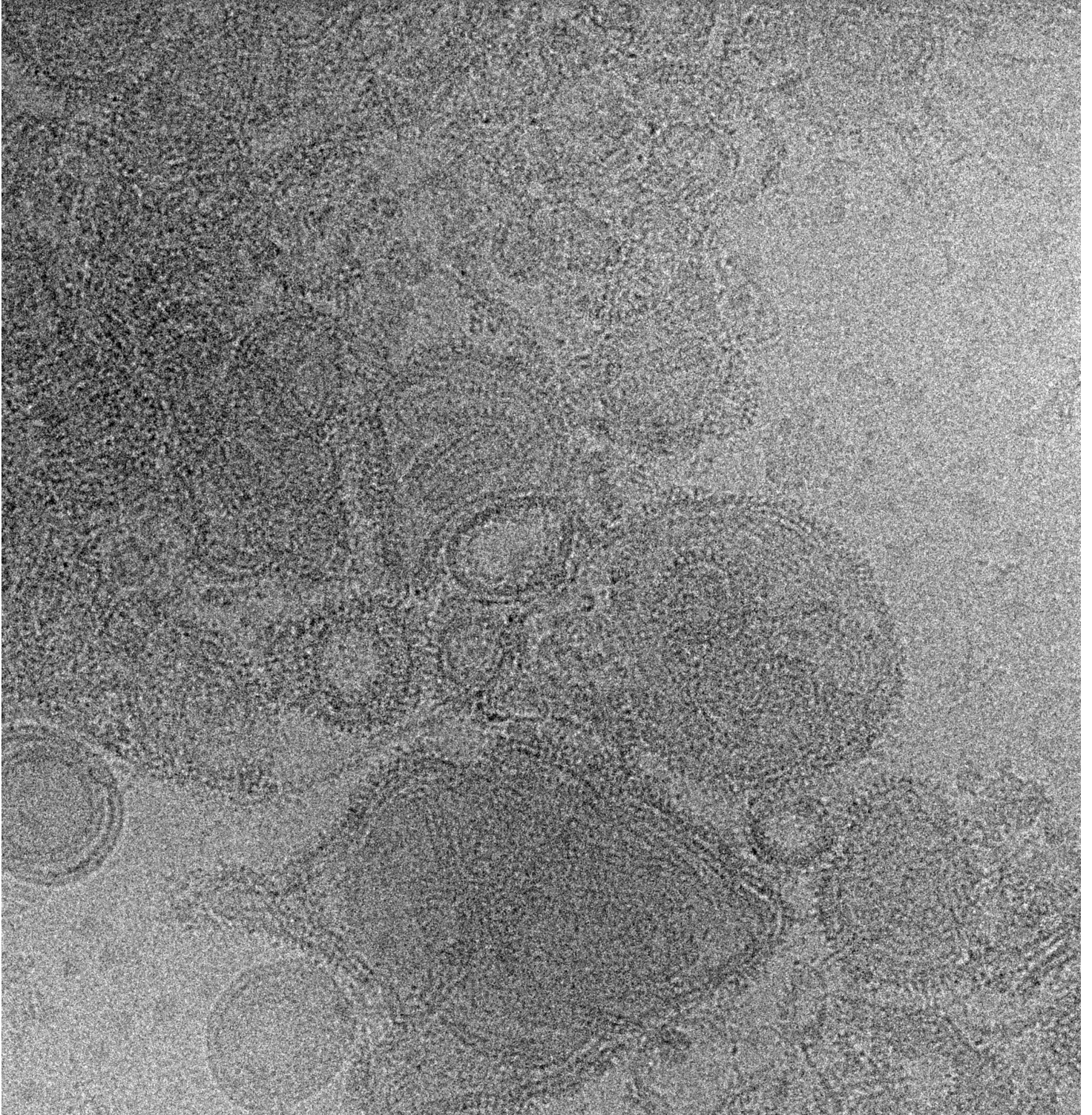

Supplement: Supplementary file 10 — Source data Fig. 6 [file 44318_2024_346_MOESM10_ESM.zip › Fig 6/6B_reght_022222_woDNA_G1_S1_Prev6_scale.pdf]

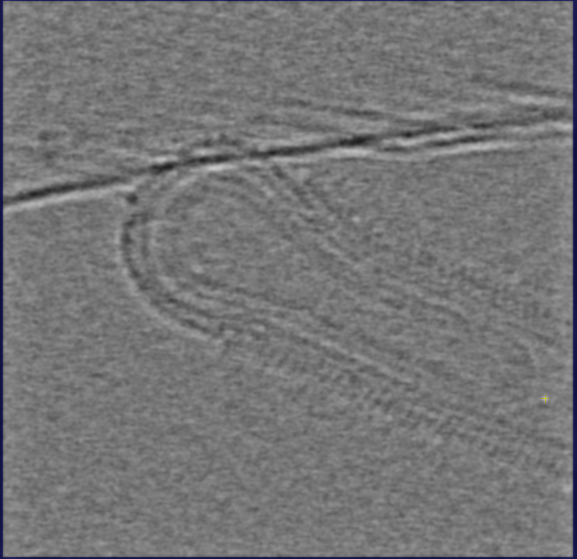

Supplement: Supplementary file 10 — Source data Fig. 6 [file 44318_2024_346_MOESM10_ESM.zip › Fig 6/6D.tif]

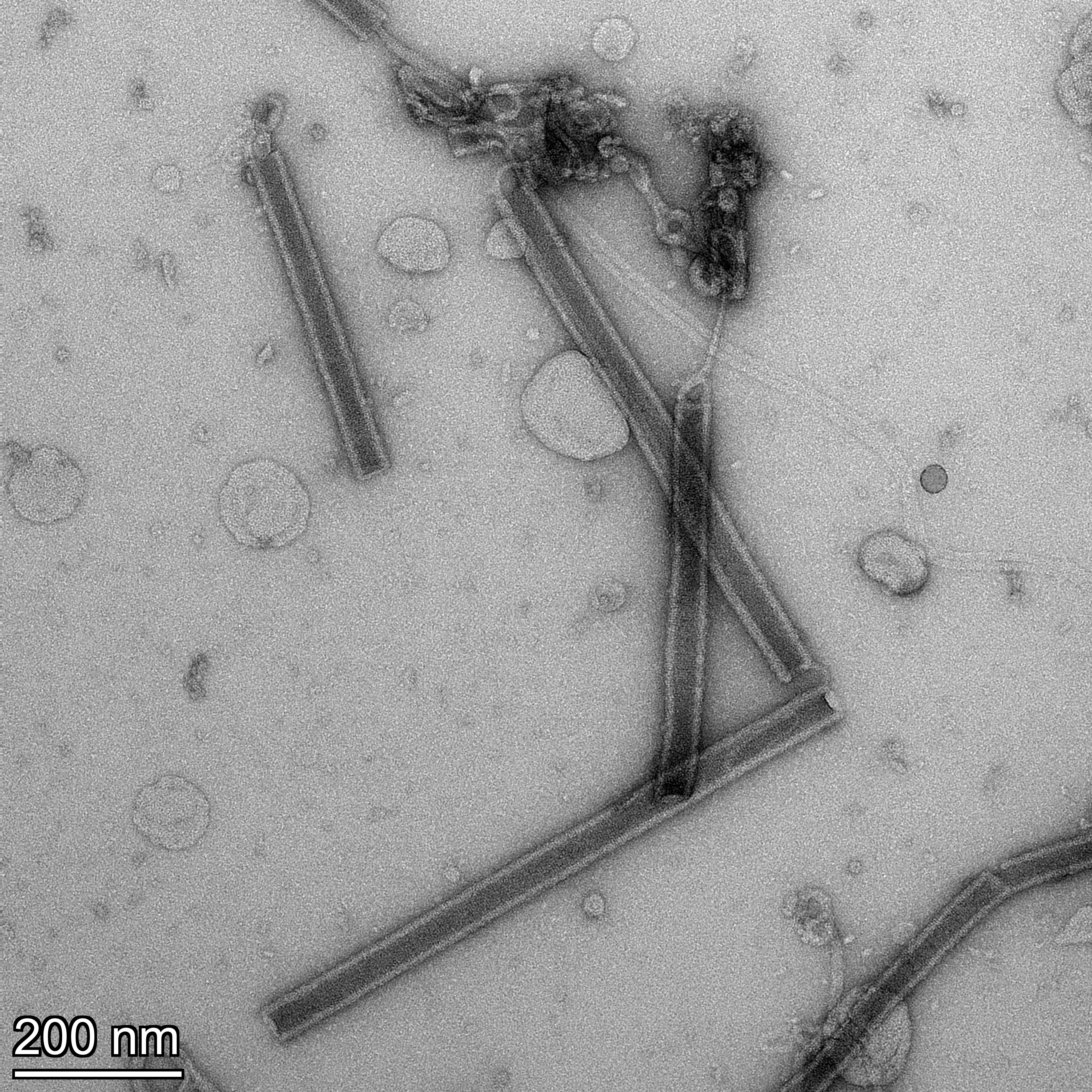

Supplement: Supplementary file 10 — Source data Fig. 6 [file 44318_2024_346_MOESM10_ESM.zip › Fig 6/6A_left.jpg]

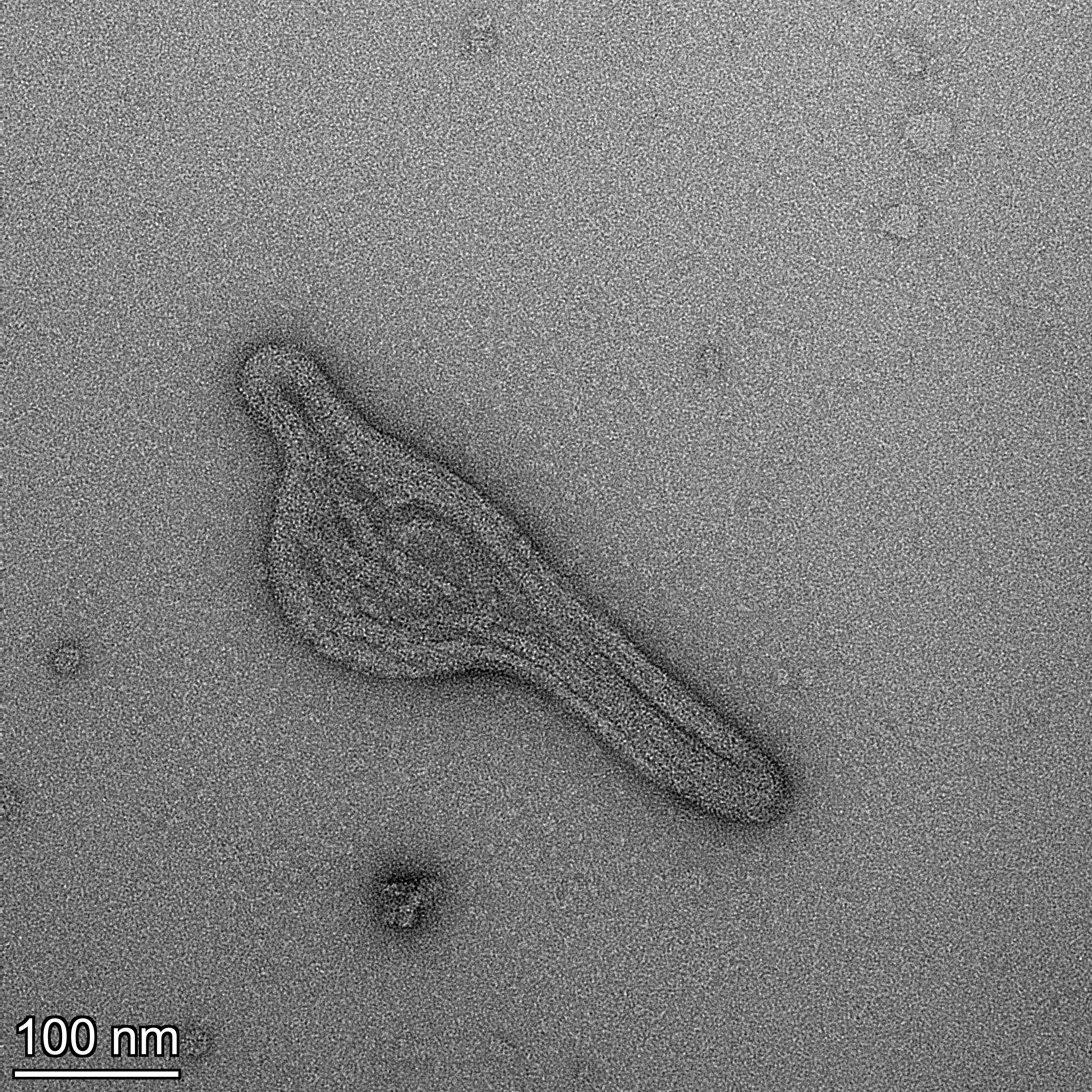

Supplement: Supplementary file 10 — Source data Fig. 6 [file 44318_2024_346_MOESM10_ESM.zip › Fig 6/6C_remodeling.jpg]

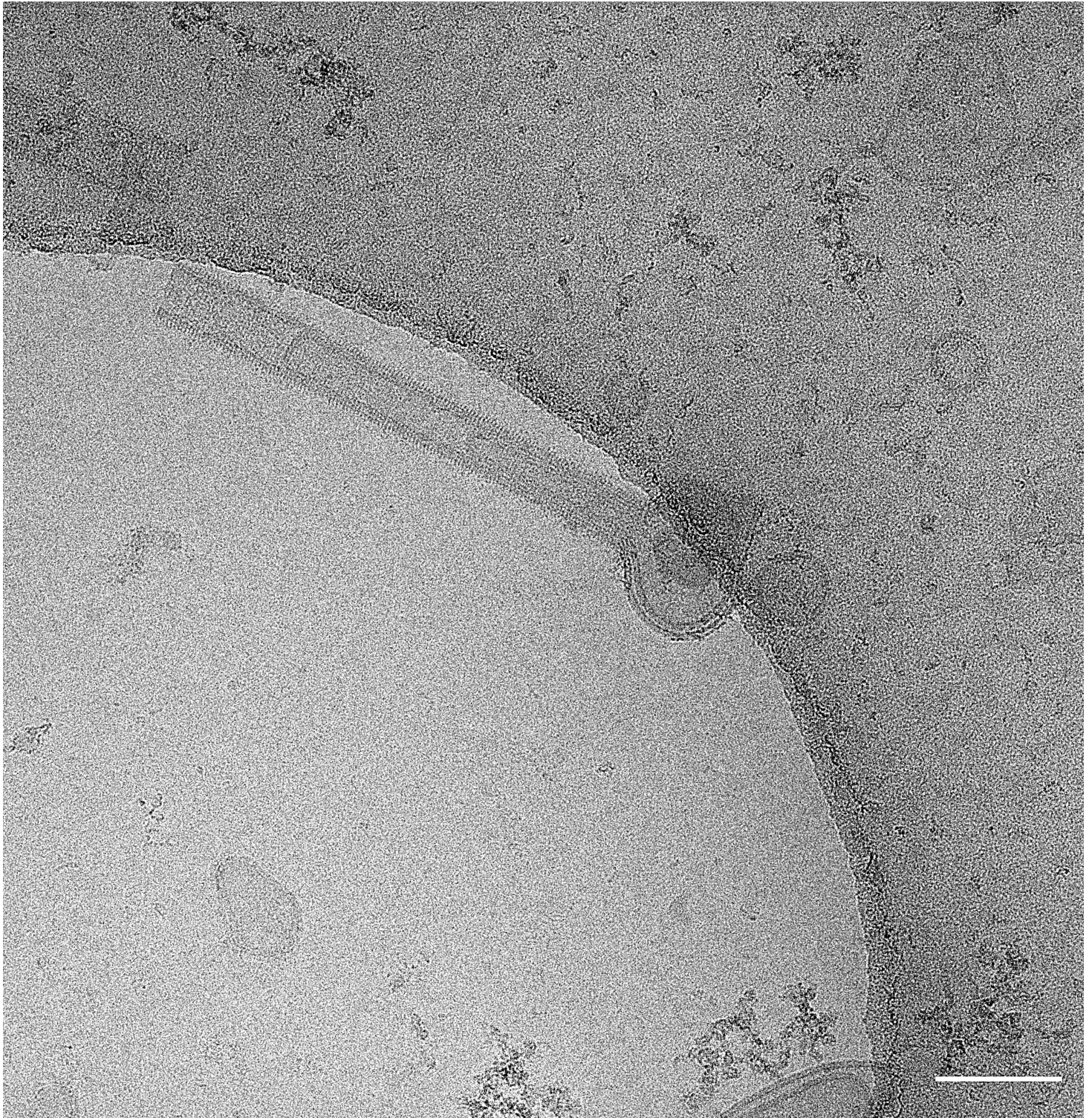

Supplement: Supplementary file 10 — Source data Fig. 6 [file 44318_2024_346_MOESM10_ESM.zip › Fig 6/6B_left_022222_wDNA_G1_S1_Prev3a_scale_v2.pdf]

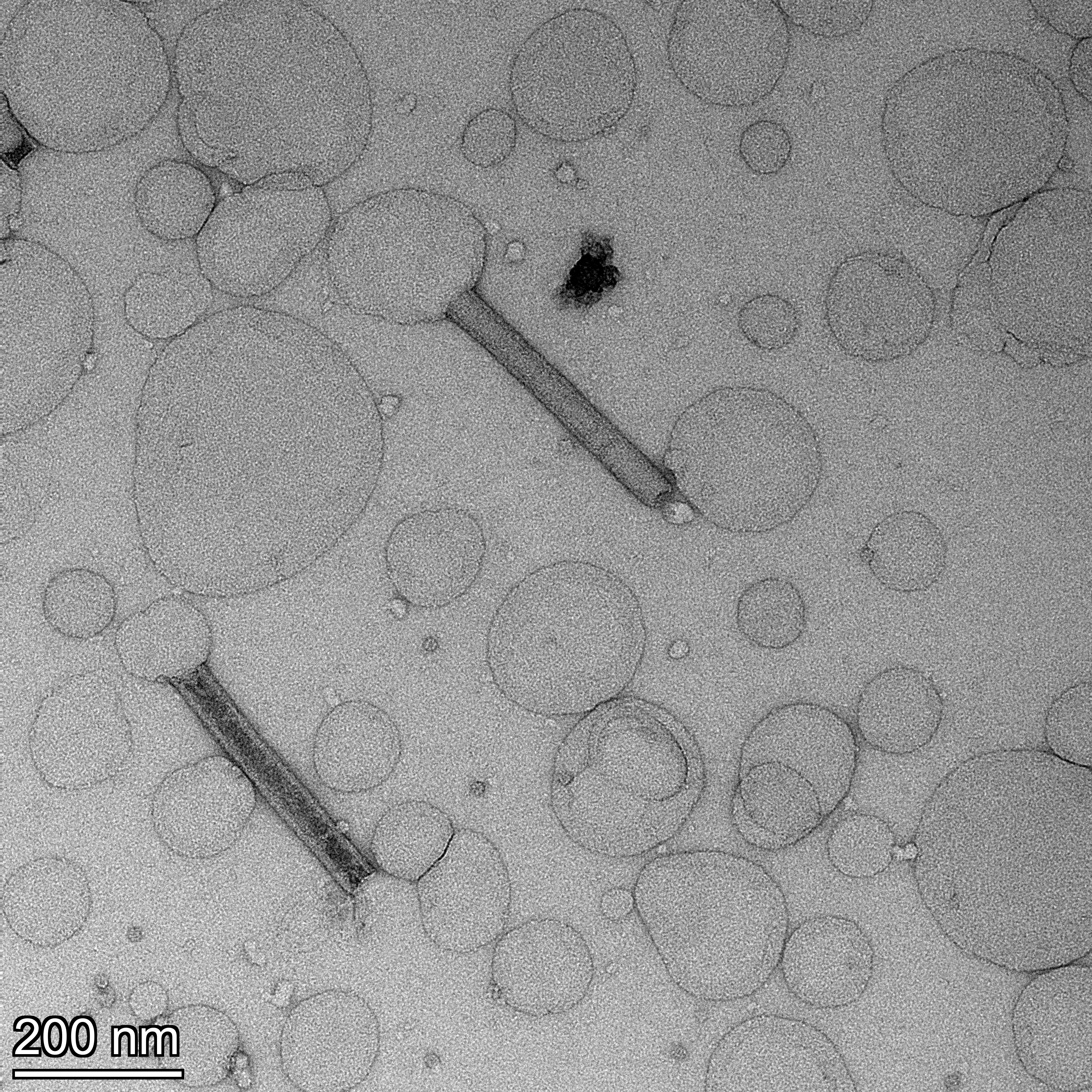

Supplement: Supplementary file 10 — Source data Fig. 6 [file 44318_2024_346_MOESM10_ESM.zip › Fig 6/6C_docking.jpg]

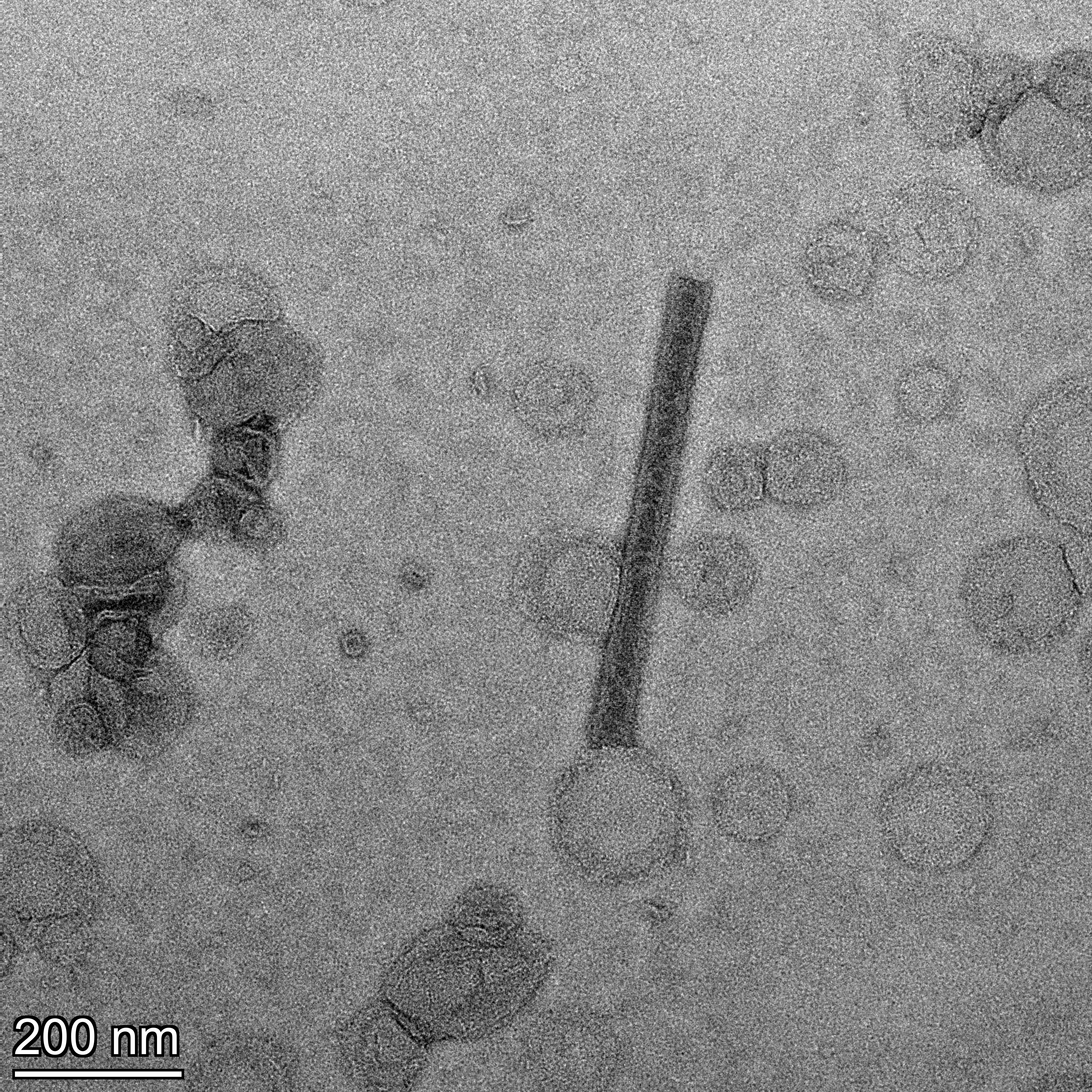

Supplement: Supplementary file 10 — Source data Fig. 6 [file 44318_2024_346_MOESM10_ESM.zip › Fig 6/6A _middle.jpg]
